# Synergistic Suppression of NF1 Malignant Peripheral Nerve Sheath Tumor Cell Growth in Culture and Orthotopic Xenografts by Combinational Treatment with Statin and Prodrug Farnesyltransferase Inhibitor PAMAM G4 Dendrimers

**DOI:** 10.3390/cancers16010089

**Published:** 2023-12-23

**Authors:** John J. Reiners, Patricia A. Mathieu, Mary Gargano, Irene George, Yimin Shen, John F. Callaghan, Richard F. Borch, Raymond R. Mattingly

**Affiliations:** 1 Institute of Environmental Health Sciences, Wayne State University, Detroit, MI 48201, USA; john.reiners.jr@wayne.edu (J.J.R.J.); p.mathieu@wayne.edu (P.A.M.); ae4865@wayne.edu (M.G.); 2 Department of Pharmacology, Wayne State University School of Medicine, Detroit, MI 48201, USA; 3 Department of Medicinal Chemistry and Molecular Pharmacology, Purdue University, West Lafayette, IN 47907, USA; george.407@osu.edu (I.G.); borch@purdue.edu (R.F.B.); 4 Currently College of Arts and Sciences, Ohio State University, Columbus, OH 43210, USA; 5 Department of Radiology, Wayne State University, Detroit, MI 48201, USA; ym_shen@wayne.edu; 6 Department of Pharmacology and Toxicology, East Carolina University, Greenville, NC 27834, USA; callaghanj22@students.ecu.edu

**Keywords:** MPNST, RAS prenylation, farnesyltransferase inhibitor, sciatic nerve

## Abstract

**Simple Summary:**

Activated RAS proteins drive the proliferation and survival of many human tumors. For example, in type 1 neurofibromatosis (NF1), loss of expression of neurofibromin allows hyper-activation of RAS. Drug treatments to block activated RAS have been long sought as a rational and targeted approach to treating cancer, but numerous obstacles have been discovered. We have designed a combinatorial approach to block the maturation of RAS in cellular models of malignant peripheral nerve sheath tumors (MPNSTs) from NF1 patients. The combination of a prodrug farnesyl transferase inhibitor (FTI) that is competitive with the prenyl co-factor of the enzyme plus a low dose of lovastatin to reduce cellular pools of prenyl precursors was effective and selective in blocking the proliferation of NF1 MPNST cells in vitro and in an orthotopic xenograft on the murine sciatic nerve. This approach could also be applicable to other cancers that are driven by activated RAS.

**Abstract:**

Neurofibromatosis type 1 (NF1) is a disorder in which RAS is constitutively activated due to the loss of the Ras-GTPase-activating activity of neurofibromin. RAS must be prenylated (i.e., farnesylated or geranylgeranylated) to traffic and function properly. Previous studies showed that the anti-growth properties of farnesyl monophosphate prodrug farnesyltransferase inhibitors (FTIs) on human NF1 malignant peripheral nerve sheath tumor (MPNST) cells are potentiated by co-treatment with lovastatin. Unfortunately, such prodrug FTIs have poor aqueous solubility. In this study, we synthesized a series of prodrug FTI polyamidoamine generation 4 (PAMAM G4) dendrimers that compete with farnesyl pyrophosphate for farnesyltransferase (Ftase) and assessed their effects on human NF1 MPNST S462TY cells. The prodrug 3-tert-butylfarnesyl monophosphate FTI-dendrimer (i.e., **IG 2**) exhibited improved aqueous solubility. Concentrations of **IG 2** and lovastatin (as low as 0.1 μM) having little to no effect when used singularly synergistically suppressed cell proliferation, colony formation, and induced N-RAS, RAP1A, and RAB5A deprenylation when used in combination. Combinational treatment had no additive or synergistic effects on the proliferation/viability of immortalized normal rat Schwann cells, primary rat hepatocytes, or normal human mammary epithelial MCF10A cells. Combinational, but not singular, in vivo treatment markedly suppressed the growth of S462TY xenografts established in the sciatic nerves of immune-deficient mice. Hence, prodrug farnesyl monophosphate FTIs can be rendered water-soluble by conjugation to PAMAM G4 dendrimers and exhibit potent anti-tumor activity when combined with clinically achievable statin concentrations.

## 1. Introduction

Type 1 neurofibromatosis (NF1) is an autosomal dominant disorder that, with a birth incidence of approximately 1 in 3000, represents the most commonly inherited cancer predisposition syndrome [1,2]. In addition to a variety of cutaneous, neurological, endocrine, cardiovascular, and orthopedic manifestations, NF1 patients typically develop one or more neurofibromas consisting of Schwann cells and associated components [3]. Approximately 10% of benign plexiform neurofibromas progress to malignant peripheral nerve sheath tumors (MPNSTs) following loss of heterozygosity at the *NF1* locus [4,5]. To date, there is no effective therapeutic approach for NF1 MPNSTs.

NF1 is caused by the loss of the tumor suppressor neurofibromin due to mutation or deletion [6,7]. Neurofibromin contains a GTPase-activating protein (GAP)-related domain (GRD) that suppresses RAS activity by driving the hydrolysis of the GTP bound to RAS [8,9]. RAS is structurally normal in NF1 tumor cells. However, loss of GRD activity in NF1 tumor cells facilitates the constitutively activated (i.e., GTP-bound) conformation of RAS and thus potentiates its functioning as an oncogene [10,11]. The significance of the loss of RAS GAP activity in NF1 growth control was emphasized by the finding that the introduction of just the neurofibromin-GRD restored normal growth to NF1 Schwann cells [12].

Prenylation entails the covalent addition of either a farnesyl or geranylgeranyl moiety onto the cysteine of the CAAX box prenylation motif. Prenylation provides an increase in hydrophobicity and perhaps additional targeting that directs translocation from the cytoplasm to membranes [13]. Ras undergoes prenylation, and this post-translation modification is needed to achieve membrane attachment and the subcellular localization required for its biological functions, including its transforming activity. Given this requirement, investigators have attempted to disrupt RAS-mediated signaling with farnesyltransferase inhibitors (FTIs). For example, in NF1, the FTI BMS-186511 inhibited the proliferation of an MPNST cell line [14], and the FTI L-739749 reduced the proliferation of neurofibromin-deficient murine Schwann cells [15]. However, no objective responses were obtained in a clinical trial in which the FTI tipifarnib was used to treat plexiform neurofibromas [16].

A caveat in the use of FTIs to modulate RAS activity stems from the findings that both K- and N-RAS can be geranylgeranylated in the presence of an FTI [17,18]. Results from our group and others indicate that N-RAS and K-RAS are the predominant active forms of RAS in a series of human NF1 MPNST cell lines [19,20]. One approach to circumventing the geranylgeranylation of RAS in the presence of an FTI could entail the use of an FTI that is competitive with the farnesyltransferase (FTase) co-substrate FPP in the presence of an HMG CoA reductase inhibitor such as lovastatin. We reasoned that suppression of HMG CoA reductase would reduce the precursor pool needed for the synthesis of the farnesyl and geranylgeranyl moieties [21]. In doing so, lovastatin co-treatment should suppress geranylgeranylation and enhance the inhibitory activity of the FTI through a reduction in the endogenous FPP pool with which the FTI would compete [22]. Preliminary studies indicated that the approach had potential [23]. Specifically, co-treatment of NF1 MPNST cell lines with a non-cytotoxic concentration of lovastatin markedly enhanced the anti-growth effects of two different prodrug *tert*-butyl farnesyl monophosphate FTIs. Although promising, an inherent limitation to the approach was the poor aqueous solubility of the prodrug FTIs.

Polyamidoamine generation 4 (PAMAM G4) dendrimers have attracted attention as vehicles for enhancing drug solubility and delivery into cells [24]. The goal of this study was to enhance the solubility of prodrug *tert*-butyl farnesyl phosphate FTIs by conjugating them to a PAMAM G4 dendrimer, which contains 64 surface primary amino groups to which moieties can be attached, and then establish their activity against NF1 MPNST cells. Conjugation of prodrug *tert*-butylfarnesyl monophosphate FTIs to PAMAM G4 dendrimers markedly enhanced the aqueous solubility of the FTIs and proved to be an effective vehicle for FTI delivery into cells. Co-treatment with clinically achievable concentrations of lovastatin markedly potentiated the anti-growth properties of the prodrug FTI dendrimer conjugates towards NF1 MPNST cells both in vitro and in vivo but had no synergistic activities on a series of normal cell types.

## 2. Materials and Methods

### 2.1. Chemicals

Lovastatin (Sigma-Aldrich, St. Louis, MO, USA) was prepared in dimethyl sulfoxide (DMSO) for cell culture experiments and stored at −80 °C. Ac-DEVD-AMC used in caspase assays was purchased from BD Biosciences (San Diego, CA, USA).

### 2.2. Cell Culture

The human NF1 MPNST cell line S462TY was the gift of N. Ratner (University of Cincinnati College of Medicine, Cincinnati, OH, USA). It was derived from a xenograft of S462 cells that grew in immune-deficient mice. The immortalized normal rat Schwann cell line iSC was provided by E. M. Shooter (Stanford University, Stanford, CA, USA). The human non-small cell carcinoma cell line A549 was obtained from Cell Lines Resource, Karmanos Cancer Institute, Detroit, MI. All three cell lines were cultured in DMEM (ThermoFisher Scientific, Waltham, MA, USA) supplemented with 10% fetal bovine serum (HyClone Laboratories, Logan, UT, USA), 100 U/mL penicillin, and 100 mg/mL streptomycin (Invitrogen, Carlsbad, CA, USA). For colony formation assays, single-cell suspensions of 700 S462TY cells, 300 iSC cells, or 1000 A549 cells were plated in 60 mm culture dishes ~18–24 h prior to treatment. The MPNST cell line ST88-14 was obtained from T. Glover (University of Michigan, Ann Arbor, MI, USA). Adherent cultures of ST88-14 cells were maintained in RPMI 1640 (Invitrogen) with 5% fetal bovine serum (HyClone Laboratories). For colony formation and MTT assays, 1000 and 2000 cells, respectively, were plated ~18–24 h prior to use. The human normal breast epithelial MCF10A cell line was obtained from Cell Lines Resource, Karmanos Cancer Institute, Detroit, MI, and cultured as previously described [25]. For MTT assays, 2500 MCF10A cells were plated per well in 96-well plates. Single-cell preparations of hepatocytes were prepared from male Sprague-Dawley rats (Harlan, Indianapolis, IN, USA) and plated at a density of 50,000 cells per well by Dr. Thomas Kocarek (Institute of Environmental Health Sciences, Wayne State University, Detroit, MI, USA). Conditions for hepatocyte isolation and culturing have been described [26]. All cultures were maintained in a humidified incubator under 5% CO_2_ at 37 °C.

### 2.3. DEVDase Activity Assay

Lysates of S462TY cultures were prepared and used for analyses of DEVDase activity as described previously [23]. Changes in fluorescence over time were converted into pmoles of product based upon comparison to a standard curve made with 7-amino-4-methylcoumarin. DEVDase-specific activities are reported as nmoles of product per minute per milligram of protein.

### 2.4. Western Blot Analyses

N-, K-, and H-RAS were detected with a 1:100 dilution of murine monoclonal pan-RAS antibody (BD Biosciences, San Diego, CA, USA, product #610002). N-RAS was detected with a 1:400 dilution of a rabbit polyclonal N-RAS antibody (Santa Cruz, CA, USA, product #sc-519). RAB5A was detected with a 1:250 dilution of a rabbit polyclonal RAB5A antibody (Santa Cruz, Santa Cruz, CA, USA, product #sc-309). Non-prenylated RAP1A was detected with a 1:250 dilution of a goat polyclonal RAP1A antibody (Santa Cruz, product #1482). GAPDH was detected with a 1:1000 dilution of rabbit polyclonal antibody to GAPDH (Cell Signaling, Boston, MA, USA, product #2118). β-actin was detected with a 1:25,000 dilution of murine monoclonal antibody to β-actin (Sigma, St. Louis, MI, USA, product #A5441). The horseradish peroxidase-conjugated secondary antibodies used were goat anti-rabbit (Pierce Biotechnology, Rockford, IL, USA, product #1858415), donkey anti-rabbit (GE Healthcare, Piscataway, NJ, USA, product #NA934V), or sheep anti-mouse (GE Healthcare, product number #NA931V). Immune complexes were visualized with an ECL detection kit (GE Healthcare, Piscataway, NJ, USA) and recorded on X-ray film.

### 2.5. Colony Formation Assays

Suspensions of S462TY, iSC, ST88-14, and A549 cells (plating densities are defined in Section 2.2) were plated in midafternoon. The following day, the plates were washed with PBS, refed, and treated in the late afternoon with control solvent, FTIs, and/or lovastatin. Thereafter, cultures were refed every 3 or 4 days until colonies were easily visible by eye. Colonies were eventually fixed with 95% ethanol, stained with crystal violet, and manually counted. All treatment groups in an experiment were processed on the same day. Colony assays employed 4 to 5 plates per treatment group.

### 2.6. MTT Assay

A slightly modified version of the original procedure described by Mosmann was used for the MTT assay [27]. Assays were performed on 96-well tissue culture plates. Cultures were generally treated ~18–24 h after plating and analyzed ~48 h after treatment. Six to seven wells were used per treatment group. Cultures were incubated in the dark for 1–3 h with the MTT reagent. The resulting formazan product was solubilized with acidified isopropanol, and the plates were read at 530 and 670 nm. The difference of OD_530_ minus OD_670_ was used as an assessment of viability.

### 2.7. Murine Sciatic Nerve Xenograft Tumor Studies and MRI Analyses

In all studies, lovastatin was first dissolved in ethanol, then treated with NaOH in order to convert it into its active form, and subsequently neutralized with HCL prior to injection into animals. Dry **IG 2** was dissolved in physiological saline and filtered through 0.45 μm sterile filters before use. Physiological saline was used to dilute both lovastatin and **IG 2** stocks.

Initial range-finding studies employed female Balb/c mice (Taconic Laboratories). Following a two-week acclimation period, mice were injected twice a day (~12 h schedule) for 7 days by i.p. injection (0.2 mL of either physiological saline or lovastatin) and/or by i.v. tail vein injection (0.3 mL of physiological saline or **IG 2**). Treatment groups are noted in the text and the legend of the figure in Section 3.6. Mice were weighed daily and euthanized within 6 to 8 h of their last treatment for tissue harvesting.

Female NOD SCID (NOD.CB17-*Prkdc^skid^*/NCrCrl) mice from Charles River Laboratories were housed in barrier facilities designed for immune-compromised animals. After a multi-week acclimation period, 25 13-week-old mice were weighed, anesthetized with isoflurane, and the ears were notched or punched to identify individual animals. Thereafter, the left leg was shaved with hair clippers to remove fur and washed sequentially with betadine and 70% alcohol. A 3/4-inch incision was made down the length of the thigh, forceps were used to tease muscle away from the sciatic nerve, and the nerve was injected with 4 microliters of suspended S469TY cells (4 × 10^4^ cells total). Following injection, the incision was closed with surgical glue, and the mice were placed on a heating pad and allowed to recover. Injections of the 25 mice occurred on two successive days. On the 21st day post-injection (having set the second day of injection as day 0), 5 mice were processed for an MRI to estimate tumor growth. On the 26th day post-injection, all 25 mice underwent MRI analyses. Thereafter, the mice were randomized and divided into 4 groups, representing treatments with (1) physiological saline by i.p. and tail vein injection, (2) lovastatin by i.p. and saline by tail vein injection, (3) **IG 2** by tail vein injection and saline by i.p. injection, or (4) lovastatin by i.p. injection and **IG 2** by tail vein injection. Doses of lovastatin (10 mg/kg) and **IG 2** (10 μmole/kg) were administered in 0.2 and 0.3 mL of physiological saline, respectively. Mice were weighed daily and treated twice a day, approximately every 12 h, for 13 days. Treatments were terminated at the end of 13 days because of developing complications with the tail vein injections and vascular collapse. MRI analyses were performed during the treatment period and 6 days after the termination of treatment.

MRI analyses of sciatic nerve tumors were performed by the Wayne State University MR Core Research Facility on a 7.0-Tesla, 30-cm bore superconducting magnet (ClinScan; Bruker, Karlsruhe, Germany) interfaced with a Siemens console (Syngo MR B15). A mouse body (transceiver) coil was used. Prior to image acquisition, anesthesia was induced by isoflurane, and the mice were kept under anesthesia throughout the acquisition time. Following a localization scan, a fat-saturation T2-weighted turbo spin echo sequence was performed for anatomical evaluation with the following parameters: Field of view, 32 mm × 32 mm; matrix size, 320 × 320; slice thickness, 0.5 mm; slice number, 20; in-plane resolution, 0.1 × 0.1 mm^2^; repetition time, 3.53 s; echo time, 56 ms; turbo factor of 7; pixel bandwidth, 130 Hz/pixel; number of average, 4. The tumor was identified by the hyper-intensity area on the T2-weighted images. Internally developed MR software SPIN (Signal Processing in MR (Version SVN Revision 1895); http://mrc.wayne.edu (accessed on 15 December 2023)) was used for tumor volume measurement.

### 2.8. General Synthetic Methods

All NMR spectra were recorded using a 300 MHz Bruker spectrometer (Billerica, MA, USA) equipped with a 5 mm multinuclear probe, unless otherwise specified. ^1^H chemical shifts are reported in parts per million from tetramethylsilane unless otherwise specified. ^31^P NMR spectra were obtained using broadband ^1^H decoupling, and chemical shifts are reported in parts per million using 1% triphenylphosphine oxide/benzene-*d*_6_ as the coaxial reference (triphenylphosphine oxide/benzene-*d*_6_ has a chemical shift of +24.7 ppm relative to 85% phosphoric acid). Mass spectral analyses were obtained from the mass spectrometry laboratory at Purdue University, West Lafayette, IN. All anhydrous reactions were carried out under an atmosphere of argon. All organic solvents were distilled prior to use, unless otherwise specified. Flash chromatography using silica gel grade 60 (230–400 mesh) was carried out for all chromatographic separations. Thin layer chromatography was performed using Analtech glass plates precoated with silica gel (250 nm). Visualization of the plates was accomplished using UV and/or Hanessian’s Stain (5.0 g of Ce(SO_4_)_2_, 25.0 g of (NH_4_)_6_Mo_7_O_24_·4H_2_O, 450 mL of H_2_O, and 50 mL of H_2_SO_4_), followed by heating. Sephadex LH-20 (GE Healthcare) was used for size exclusion-based separations.

**Figure 1:**
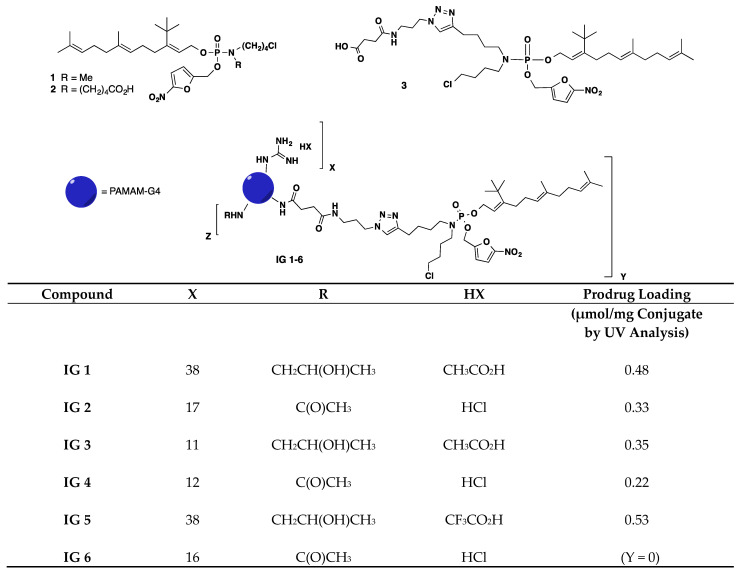
Structures of prodrug farnesyl transferase inhibitors (FTIs), dendrimers, and prodrug FTI PAMAM G4 dendrimers.

The syntheses of farnesyl monophophate prodrugs FTI-1 and FTI-2 (labeled as **1** and **2**, respectively; Figure 1) have been previously described [23,28]. Figure 1 also provides the structures of the 6 prodrug FTI PAMAM G4 dendrimers (**IG 1–6**) examined in our study. The detailed syntheses and chemical characterizations of the prodrug FTIs and prodrug PAMAM G4 dendrimers analyzed in this study are provided in the Synthetic Procedures document in the Supplementary Materials. This document also includes Scheme S1, which outlines the synthesis of the compounds in this study, plus an alternate procedure for the scaled-up synthesis of gram quantities of **IG 2**.

### 2.9. Analyses of Synergism and Statistical Significance

Drug–drug interactions were analyzed with CompuSyn software Version 1.0 (Informer Technologies, Los Angeles, CA, USA; software is available at https://www.compusyn.software.informer.com (accessed on 27 September 2023)) using plots of combination index (CI) versus fraction affected (Fa). Effects on cell viability/proliferation as recorded by MTT assays were determined by first establishing concentration–response curves with 5 to 6 concentrations of lovastatin and **IG 2**, followed by analyses of effects with 3 to 5 concentrations of **IG 2** at a fixed concentration of lovastatin. In vivo S462TY sciatic nerve tumor xenograft data were analyzed using IBM SPSS Statistics software version 29.0.1.0 (IBM, Armonk, NY, USA). The data were checked for homogeneity of variances using Levine’s test. The combination therapy was then compared to the control using a one-tailed welch *t*-test. A *p* value < 0.05 was considered statistically significant.

## 3. Results

### 3.1. Antiproliferative Properties of Farnesyl Monophosphate Prodrug Dendrimer Analogs

**Figure 2:**
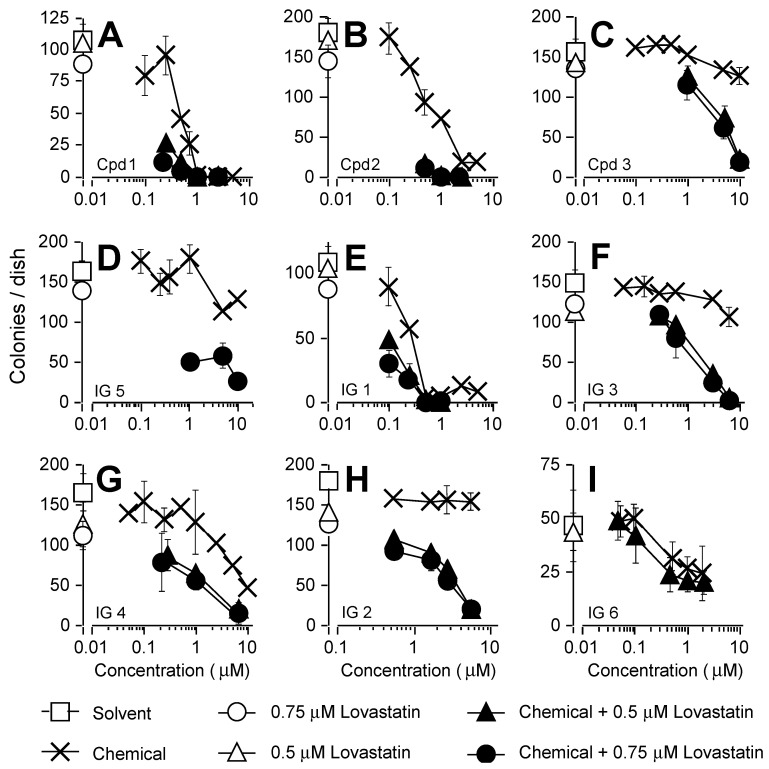
Suppression of colony formation by prodrug FTIs (panels **A**–**C**), prodrug FTI PAMAM G4 dendrimers (panels **D**–**H**), and PAMAM G4 dendrimer lacking conjugated FTI (panel **I**). Overnight cultures of S462TY cells were treated with DMSO, 0.5 or 0.75 μM of lovastatin, or varied concentrations of a prodrug FTI, dendrimer, or prodrug FTI dendrimers. After four days of treatment, the cultures were washed and refed with fresh medium lacking any drugs. Cultures were fixed and stained 5 days later for assessment of colony formation. Treatments are noted in the figure legend. All data represent the means ± SD of 4–5 plates per treatment group. The data are representative of a minimum of 2 to 4 independent experiments.

We previously reported that the *tert*-butylfarnesyl monophosphate prodrug FTIs **1** and **2** (Figure 1) were cytotoxic to the NF1 MPNST cell lines NF90-8 and ST88-14 when used in combination with lovastatin [23,29]. Both prodrugs, in the absence of lovastatin, suppressed colony formation by the NF1 MPNST cell line S462TY when used at ≥0.3 μM (Figure 2A,B). Lovastatin by itself, at either 0.5 μM or 0.75 μM, had minimal effects on colony formation (Figure 2A,B). However, the suppressive effects of the two FTIs were greatly enhanced by combinational treatment with lovastatin (Figure 2A,B).

Both **1** and **2** have low solubility in aqueous solvents. In order to circumvent this limitation, we conjugated prodrug *tert*-butylfarnesyl monophosphate FTIs to the PAMAM G4 dendrimer. Most of the prodrug FTI dendrimers we tested were based on conjugates of PAMAM G4 and FTI **3** (Figure 1). The latter is a derivative of **2** that contains a ‘linker’ sequence that facilitates its attachment to any of the 64 surface amine groups on the PAMAM G4 dendrimer. FTI **3** was also fairly insoluble in the aqueous solvent and less potent than **2** at suppressing S462TY colony formation by itself. However, its suppressive effects were markedly enhanced by co-treatment with lovastatin (Figure 2C).

A series of FTI **3**-based prodrug PAMAM G4 dendrimers were synthesized that differed in the number of attached FTI prodrug, guandinium, and capping moieties (see Figure 1). The FTIs **IG 1** and **IG 5** had an identical number of guanidinium groups (i.e., 38) and approximately the same number of prodrug FTI moieties per dendrimer. By itself, **IG 5** exhibited a concentration-dependent suppression of colony formation that was similar to **3** (Figure 2D), whereas **IG 1** exhibited greater potency and efficacy (Figure 2E). Co-treatment with lovastatin enhanced the suppressive effects of both **IG 5** and **IG 1** (Figure 2D,E). Prodrug FTI dendrimers **IG 3** and **IG 4** contained a similar number of guanidium residues (11 and 12, respectively). Both FTI dendrimers were very insoluble in aqueous solvents. However, both FTI conjugates after solubilization in DMSO, when used in combination with lovastatin, exhibited combinational potencies similar to those of non-conjugated prodrug **3** (compare Figure 2C with Figure 2F,G).

FTI dendrimer **IG 2** contained an intermediate number of guanidine residues (i.e., 17; see Figure 1) and was soluble in aqueous solvents at 35 mg/mL. By itself, **IG 2** had no effect on S462TY colony formation up to 7 μM (Figure 2H). Co-treatment with lovastatin markedly enhanced **IG 2** suppression of colony formation. This potentiation was observed with concentrations of **IG 2** as low as 0.5 μM (Figure 2H).

Compound **IG 6** is a PAMAM G4 dendrimer containing 16 guandinium moieties (similar to **IG 2**) but no prodrug FTI moieties (Figure 1). It was used to assess if the dendrimer component of **IG 2** contributed to the observed potentiation following **IG 2** and lovastatin co-treatment. By itself, **IG 6** exhibited a weak suppressive effect on colony formation (Figure 2I). This may reflect the greater number of acetyl moieties used for blocking the cationic surface amines compared to the prodrug FTI dendrimers. Nevertheless, co-treatment with lovastatin did not enhance the suppressive effects of **IG 6** (Figure 2I). It should be noted that the concentrations reported in Figure 2D–H represent FTI prodrugs. If **IG 6** and the prodrug FTI dendrimers reported in Figure 2D–H were compared on the basis of dendrimer content, the **IG 6** curve in Figure 2H would have to be shifted to the right by a factor of 2 to 3 (which varies depending on the prodrug FTI being compared).

### 3.2. Analyses of ***IG 2*** Effects on S462TY Proliferation, Viability, and Caspase Activation

**Figure 3:**
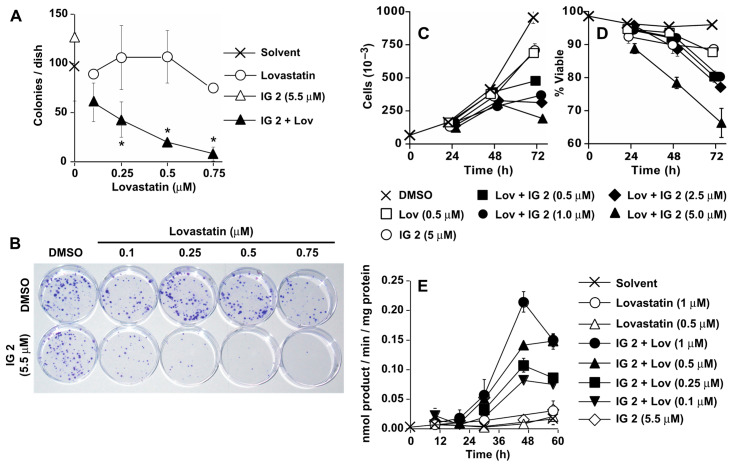
Suppression of S462TY proliferation in culture by **IG 2** and lovastatin co-treatment. In all panels, overnight cultures of S462TY cells were treated with DMSO, **IG 2**, lovastatin, or varied concentrations of lovastatin ± **IG 2**. Treatments and concentrations are noted in each panel. (**A**,**B**) After four days of treatment, cultures were washed and refed with fresh medium lacking any drugs. Cultures were fixed and stained 5 days later for colony counting (**A**) and photographing (**B**). The data in (**A**) represent the means ± SD of 4 plates per group and are representative of 3 independent experiments. * Significantly less than values for cultures treated with only solvent, lovastatin, or **IG 2**, *p* < 0.05. (**C**,**D**) Overnight cultures were treated as indicated and subsequently treated with trypsin at the indicated times for estimates of total cell count (**C**) and the percentage of trypan blue non-permeable (viable) cells (**D**). The percent of viable cells was calculated as the number of trypan blue impermeable cells divided by the sum of trypan blue permeable and non-permeable cells multiplied by 100. The data represent the means ± SD of 4 plates per treatment. (**E**) Overnight cultures were treated as indicated and harvested at varied times for analyses of DEVDase activities (measure of procaspase-3/7 activation). The data represent the means ± SD of triplicate analyses.

We selected **IG 2** for further analyses because of its solubility properties and its potency and efficacy at inhibiting colony formation when used in combination with lovastatin. Concentrations of lovastatin as low as 250 nM reproducibly enhanced **IG 2** suppression of colony formation (Figure 3A,B). To delineate whether the reductions in colony formation represented a cytostatic and/or cytotoxic effect, cultures were treated with trypsin, incubated with trypan blue, and then counted. Trypan blue permeable cells were considered to be non-viable. Lovastatin and **IG 2** at concentrations of 0.5 μM and 5 μM, respectively, had only minor effects on proliferation or viability (Figure 3C,D). However, co-treatment with 0.5 μM lovastatin and varied concentrations of **IG 2** resulted in concentration-dependent decreases in cell number (Figure 3C) and, to a much lesser degree, losses in viability (Figure 3D; note the scale for viability).

Although the contribution of cytotoxicity to the overall anti-proliferative activity of combinational treatment appeared to be minor, some S462TY cells in co-treated cultures developed apoptotic features (e.g., shrunken cells decorated with small membrane blebs). The development of these morphological features was accompanied by increases in DEVDase activity, a measure of procaspase-3/7 activation (Figure 3E). Neither lovastatin (as high as 1 μM) nor **IG 2** (5.5 μM) increased DEVDase-specific activities above those measured in the solvent controls over a 58-h period. However, combining **IG 2** with concentrations of lovastatin as low as 0.1 μM caused a concentration-dependent activation of DEVDase that paralleled reductions in colony formation (compare Figure 3A with Figure 3E).

### 3.3. Analyses of Synergy in Co-Treatment Protocols with S469TY Cells

**Figure 4:**
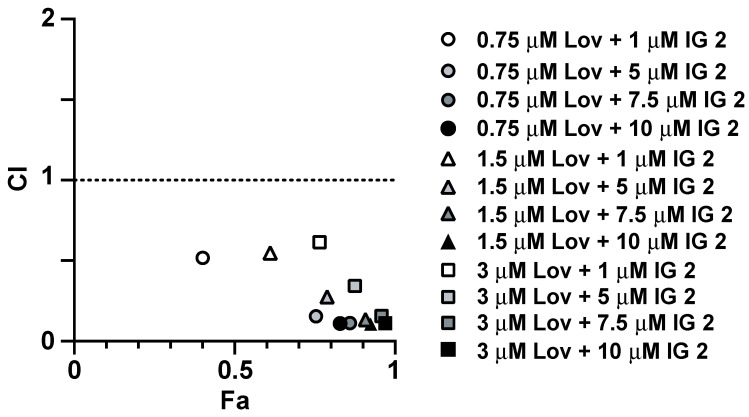
Synergistic inhibition of S462TY proliferation by combinational lovastatin plus **IG 2** treatment. Overnight cultures of S462TY cells plated in 96-well culture plates were treated with six different concentrations of lovastatin and **IG 2** for 48 h before being processed for MTT assays in order to establish concentration response curves. Based upon these data, three fixed concentrations of lovastatin (0.75, 1.5, and 3 μΜ) were incubated with varied concentrations of **IG 2** (1, 5, 7.5, and 10 μM) for 48 h before being processed for MTT assays. The resulting data were used to construct CI vs. Fa plots. Data points below a CI value of 1 represent a synergistic interaction. MTT assays entailed 6–7 wells per treatment.

Both the colony formation (Figure 3A,B) and cell counting (Figure 3C) studies suggest a cooperative effect in co-treated cultures. To investigate this relationship in further detail, we measured the effects of four varied concentrations of **IG 2** with three fixed concentrations of lovastatin on S462TY viability/proliferation in an MTT assay (Figure 4). Plots of combination index (CI) versus fraction affected (Fa) can be used to assess antagonistic, additive, and synergistic outcomes in combinational treatment protocols [30]. Data points that fall below the y-axis value of 1 in CI vs. Fa plots represent a synergistic interaction. Synergy was observed at all 4 concentrations of **IG 2** with each of the 3 lovastatin concentrations (Figure 4).

### 3.4. Analyses of Effects of Co-treatment on Proliferation/Viability in Other MPNST and Normal Cells

**Figure 5:**
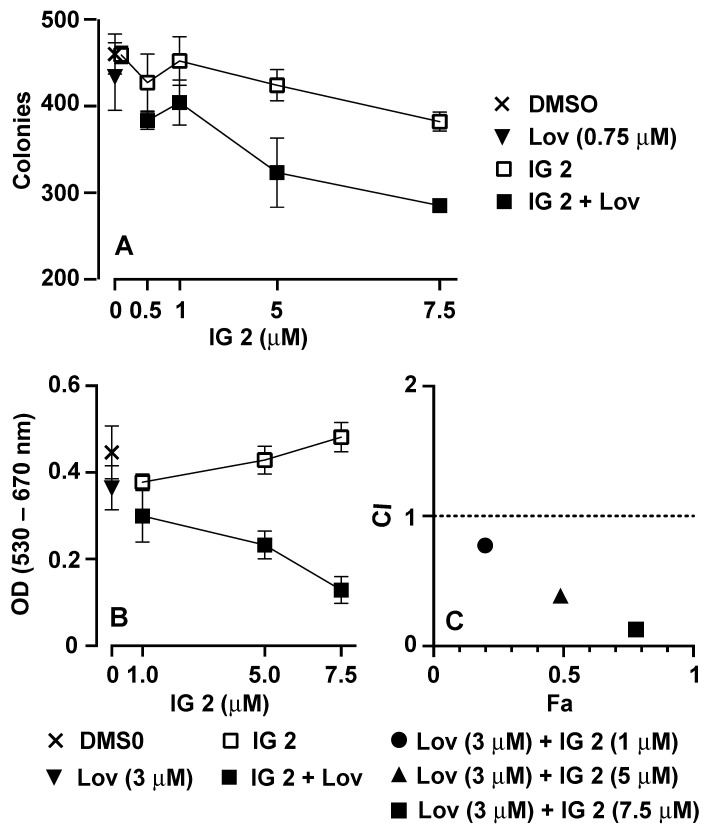
Synergistic inhibition of MPNST ST88-14 cell proliferation by combinational lovastatin and **IG 2** treatment. In all panels, overnight cultures of ST88-14 cells were treated with DMSO, **IG 2**, lovastatin, or varied concentrations of lovastatin ± **IG 2**. Treatments and concentrations are noted in each panel. (**A**) After four days of treatment, cultures were washed and refed with fresh medium lacking any drugs. Cultures were fixed and stained 5 days later for colony counting. The data represent the means ± SD of 4 plates per group. (**B**) Overnight cultures in 96-well plates were treated as indicated for two days before being processed for MTT assays. (**C**) Overnight cultures plated in 96-well culture plates were treated with six different concentrations of lovastatin and **IG 2** for 48 h before being processed for MTT assays in order to establish concentration response curves. Based upon these data, a fixed concentration of lovastatin (3 μM) was incubated with 1, 7.5, or 10 μM of **IG 2** for 48 h before being processed for MTT assays. The resulting data were used to construct CI vs. Fa plots. Data points below a CI value of 1 represent synergism.

NF1-derived MPNST ST88-14 cells express activated Ras [19,20]. Co-treatment of ST88-14 cells with concentrations of lovastatin or **IG 2** that had little to no effect individually resulted in a cooperative inhibition of colony formation (Figure 5A). MTT assays also show a cooperative inhibition of growth/viability by lovastatin plus **IG 2** co-treatment (Figure 5B). CI versus Fa plots of MTT-derived data indicated that lovastatin and **IG 2** worked synergistically in inhibiting the proliferation/viability of ST88-14 cultures (Figure 5C).

**Figure 6:**
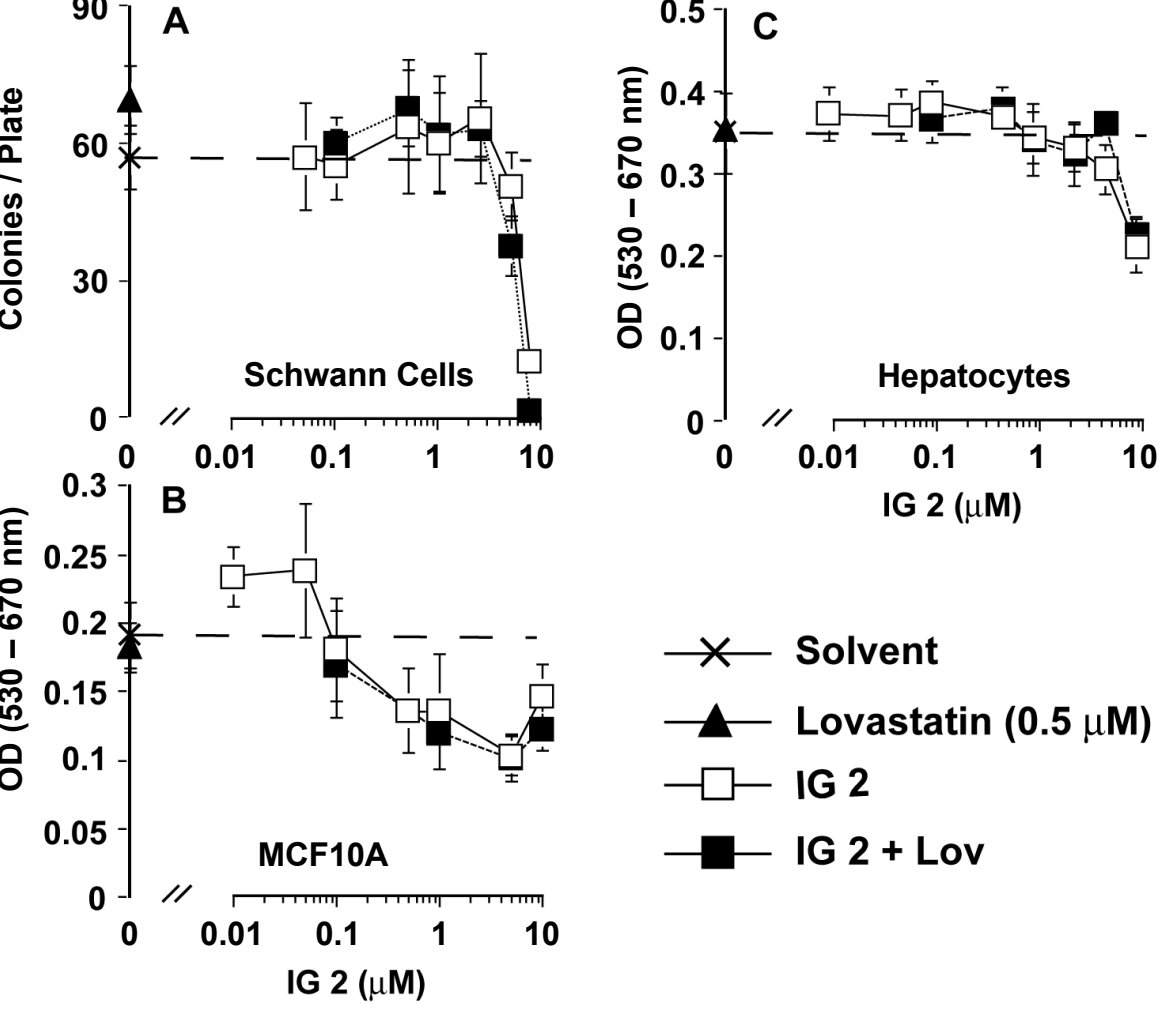
Effects of **IG 2** and lovastatin co-treatment on the growth/viability of non-tumor cells. (**A**) Overnight cultures of immortalized, non-tumorigenic rat iSC cells were treated with DMSO, 0.5 μM of lovastatin, or varied concentrations of **IG 2** ± lovastatin. After five days, the cultures were washed and refed with fresh medium. The cultures were subsequently refed a second time and harvested for staining and counting 3 days later. The data represent the means ± SD of 5 culture dishes per treatment group and are representative of two independent experiments. Cultures of human breast MCF10A epithelial cells (**B**) and primary rat liver hepatocytes (**C**) were treated as described in (**A**) and processed for MTT assays 48 h later. The data in panels (**B**,**C**) represent the means ± SD of 6 culture wells per treatment and are representative of two independent experiments with each cell type. The dashed line in the panels represents the value of the DMSO control.

iSC cells are a non-tumorigenic, immortalized rat Schwann cell line. Neither 0.5 μM of lovastatin nor concentrations of **IG 2** up to 5 μM affected iSC colony formation (Figure 6A). However, unlike the results obtained with the S462TY cell line, we observed no synergistic suppression of iSC colony formation following co-treatment with lovastatin and **IG 2** (Figure 6A). Similarly, although concentrations of **IG 2** could be reached that were cytostatic/cytotoxic (as scored in an MTT assay) to cultures of normal human breast MCF10A epithelial cells (Figure 6B) and primary rat hepatocytes (Figure 6C), co-treatment with lovastatin did not enhance the effects of **IG 2**.

### 3.5. Lovastatin and FTI Prodrug Dendrimer Effects on Prenylation

**Figure 7:**
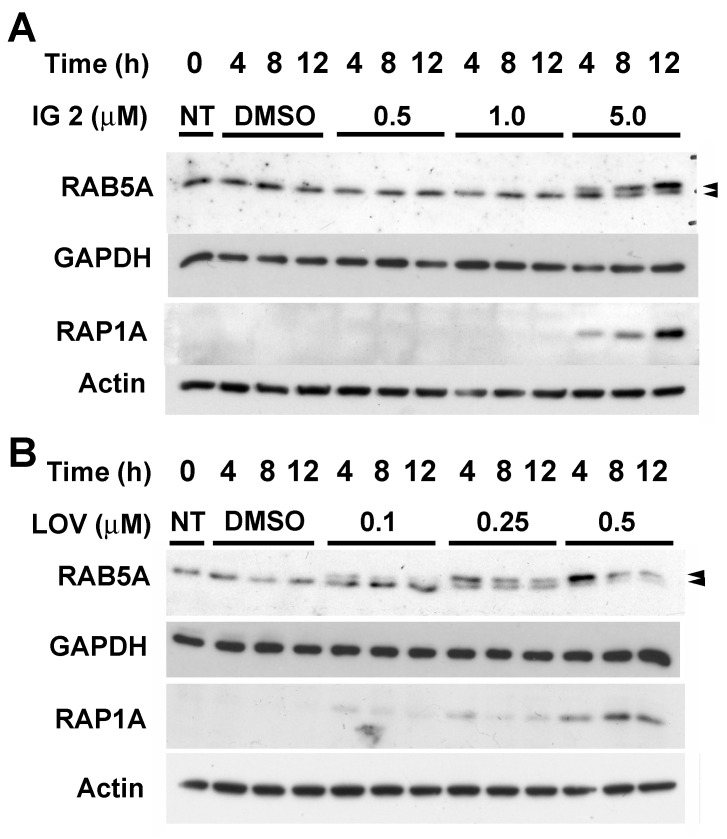
Effects of **IG 2** and lovastatin on RAB5A and RAP1A prenylation. Two-day-old S462TY cultures were left untreated or treated with DMSO or varied concentrations of either **IG 2** (**A**) or lovastatin (**B**) before being harvested for western blot analyses of RAB5A and RAP1A. The antibody used for RAP1A detects the nonprenylated form of the protein. The upper of the two bands detected with anti-RAB5A represents non-prenylated RAB5A. Each lane contained 25 μg of protein. Similar results were obtained in a second independent experiment.

In the initial studies, we attempted to define the concentration range over which **IG 2** and lovastatin affected prenylation in S462TY cells. RAP1A and RAB5A are geranylgeranylated and considered representative substrates of GGTase I and GGTase II, respectively [31]. N- and K-RAS can be farnesylated and geranylgeranylated by FTase and GGTase I, respectively [31]. Cultures exposed to 5 μM of **IG 2** (but not 0.5 or 1.0 μM) accumulated non-prenylated RAB5A (upper band, Figure 7A and Figure S1) and non-prenylated RAP1A (antibody recognizes only the non-prenylated form, Figure 7A and Figure S2). These accumulations occurred within 4 h of treatment and increased with passing time. Accumulations of non-prenylated RAB5A were also observed within 4 h of treatment with 0.25 μM of lovastatin (Figure 7B and Figure S3). Treatment with a higher concentration of lovastatin (0.5 μM) accelerated the accumulation of non-prenylated RAB5A. Treatment with 0.5 μM of lovastatin also stimulated the accumulation of non-prenylated RAP1A (Figure 7B and Figure S4).

**Figure 8:**
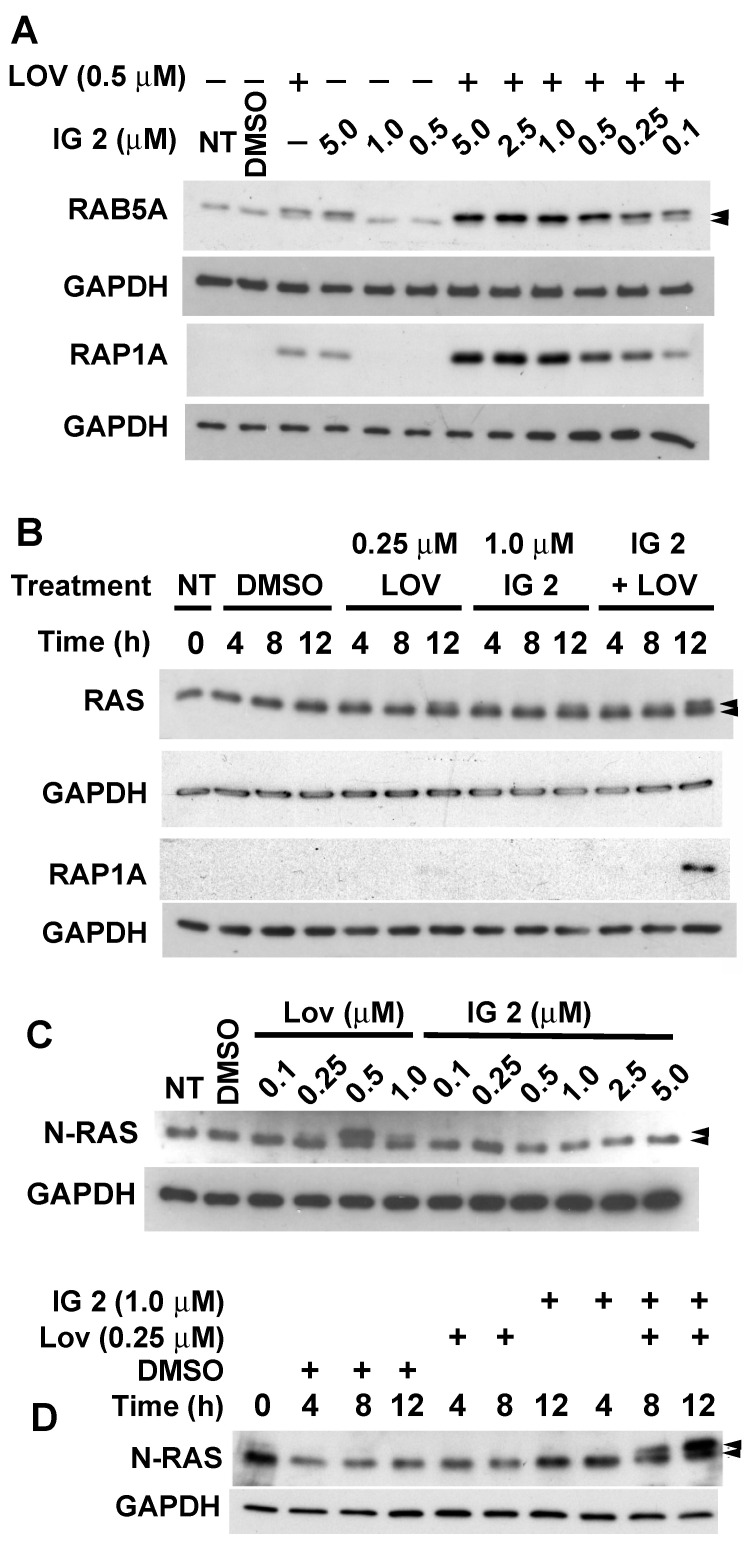
Combinational effects of **IG 2** and lovastatin on prenylation. S462TY cultures were left untreated (NT) or treated with DMSO, fixed or varied concentrations of only lovastatin or **IG 2**, or combinations of lovastatin and **IG 2**, as indicated in the individual panels. Cultures were harvested 12 h after treatment (**A**,**C**) or at varied times after treatment (**B**,**D**) for subsequent western blot analyses of prenylation. The upper of the two bands detected with antibodies to pan-RAS, N-RAS, and RAB5A represent the non-prenylated protein. The antibody used for the identification of RAP1A recognizes only the non-prenylated protein. Each lane contained 25 μg of protein.

Lovastatin (0.5 μM) markedly increased the accumulation of non-prenylated RAB5A and RAP1A in cultures treated with varying concentrations of **IG 2** (Figure 8A, Figure S5 and Figure S6). This enhancement occurred with **IG 2** concentrations as low as 0.25 μM and was maximal at 1 μM (Figure 8A, Figure S5 and Figure S6). If the **IG 2** concentration was fixed at 1 μM and the lovastatin concentration was further reduced to 0.25 μM, we could clearly detect an enhanced accumulation of non-prenylated RAP1A (Figure 8B and Figure S7). Minor accumulations of non-prenylated pan RAS were observed in cultures treated singularly for 12 h with either 0.25 μM of lovastatin or 1 μM of **IG 2** (upper band, Figure 8B and Figure S8). In co-treated cultures, however, comparable accumulations of non-prenylated pan RAS occurred earlier (i.e., within 4 h of treatment) and continued to increase over a 12-h period (Figure 8B and Figure S8). Analyses of specifically N-RAS demonstrated that treatment for 12 h with 0.5 μM, but not 0.1 or 0.25 μM of lovastatin, caused an accumulation of non-prenylated N-RAS (Figure 8C and Figure S9). Concentrations of **IG 2** as high as 5 μM had no effect on N-RAS prenylation (Figure 8C and Figure S9). However, when S462TY cultures were co-treated with concentrations of lovastatin (0.25 μM) and **IG 2** (1.0 μM) that singularly had no effect on N-RAS prenylation, dramatic accumulations of non-prenylated RAS were observed within 4 h of co-treatment and continued to increase with time (Figure 8D).

### 3.6. In Vivo Toxicity Studies

In the initial studies, we determined that twice daily dosing of female Balb/c mice with ~100 μmole/kg of **IG 2** per injection, or 40 mg/kg of lovastatin + 20 μmole/kg of **IG 2**, was either lethal or necessitated euthanasia of treated mice within a three-day period. With twice daily 20 mg/kg of lovastatin + 20 μmole/kg of **IG 2** treatments, two out of four mice exhibited hind limb paralyses that necessitated termination of treatment. Subsequent studies indicated that twice daily treatment, for seven consecutive days, with 10 or 20 mg/kg of lovastatin, 20 μmole/kg of **IG 2**, 20 mg/kg of lovastatin + 10 μmole/kg of **IG 2**, or 10 mg/kg of lovastatin + 20 μmole/kg of **IG 2** had no adverse effects on mouse weights, general appearance, or mobility of the mice.

**Figure 9:**
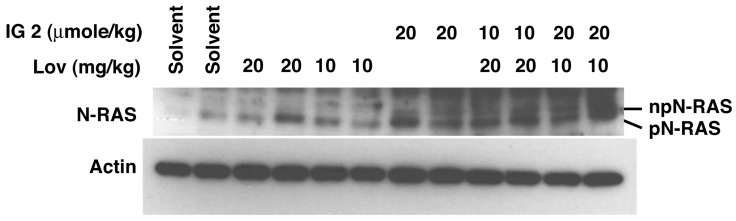
In vivo effects of lovastatin and **IG 2** on N-Ras prenylation. Female Balb/c mice were treated twice a day, for 7 days, with i.p. plus i.v. injections of physiological saline, i.v. injections of physiological saline plus i.p. injections of 10 or 20 mg/kg of lovastatin, i.p. injections of physiological saline plus i.v. injections of 20 μmole/kg of **IG 2**, or i.p. injections of 10 or 20 mg/kg of lovastatin plus i.v. injections of 10 or 20 μmole/kg of **IG 2**. Mice were euthanized 6–8 h after the final treatment for harvesting of lung tissues and analyses of prenylated (pN-Ras) and non-prenylated N-Ras (npN-Ras). Each lane contains 25 μg of protein and represents tissue isolated from an individual mouse.

We were unable to isolate sufficient amounts of protein from sciatic nerves for western blot analyses of protein prenylation. Hence, we analyzed lung tissue because of its abundance and facile exposure to i.v. administered **IG 2**. We did not detect non-prenylated N-Ras in lung tissue harvested from mice treated twice daily for 7 days with either physiological saline or 10 or 20 mg/kg of lovastatin (Figure 9A and Figure S10). A small percentage of lung N-Ras was non-prenylated following twice-daily treatment with 20 μmol/kg of **IG 2** (Figure 9 and Figure S10). Co-treatment with 10 mg/kg of lovastatin markedly increased the amount of non-prenylated N-Ras (Figure 9 and Figure S10).

### 3.7. Orthotopic Sciatic Nerve S469TY Xenograft Studies

**Figure 10:**
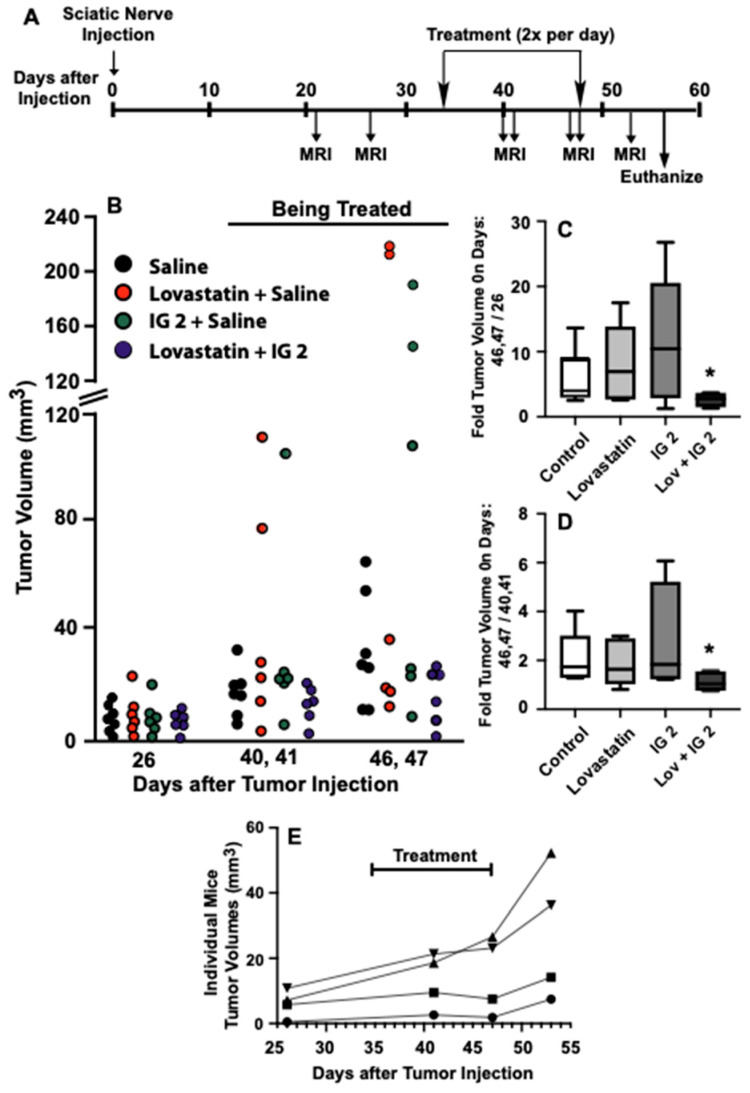
Suppression of S462TY sciatic nerve xenograft growth by combinational lovastatin and **IG 2** treatment. (**A**) Diagram of the tumor treatment protocol. The left sciatic nerves of 25 female NOD SCID mice were injected with suspensions of S462TY cells at time zero. The xenograft’s development was monitored by MRI. Mice were treated twice a day by i.v. and i.p. with saline, or i.v. with 10 μmole/kg of **IG 2** and i.p. with saline, or i.v. with saline and i.p. with 10 mg/kg of lovastatin, or i.v. with **IG 2** and i.p. with lovastatin. (**B**) Volumes of individual sciatic nerve xenografts during different stages of the study. (**C**) Box plots of the ratios of individual tumor volumes at the end of treatment (days 46,47) by their volumes prior to treatment (day 26). * Significantly less than the control group, *p* < 0.05. (**D**) Box plots of the ratios of individual tumor volumes at the end of treatment (days 46,47) by their volumes halfway through treatment (days 40,41). * Significantly less than the control group, *p* < 0.05. (**E**) Tumor rebound in the **IG 2** and lovastatin combinational treatment group following termination of treatment. The symbols identify 4 mice whose tumors were monitored over the time course.

An orthotopic sciatic nerve xenograft model was employed to assess the effects of combinational lovastatin and **IG 2** treatment on the growth of established S462TY tumors growing on the sciatic nerves of immune-compromised NOD SCID mice. The protocol we employed is depicted in Figure 10A and entailed twice-daily treatment of mice bearing established S462TY sciatic nerve xenografts with solvent, lovastatin, **IG 2**, or a combination of the two agents. The doses of **IG 2** and lovastatin we employed were based on the in vivo studies with Balb/c mice. Tumor growth was monitored by MRI. An example of the xenografts that developed following injection of S462TY cells and the MRI images collected for estimation of tumor volume are presented in Supplementary Material Figure S11.

Tumor volumes prior to the initiation of treatment, during treatment, and at the end of treatment are depicted in Figure 10B. Tumor volumes increased with time in all 4 treatment groups, although only marginally in mice treated with the combination of lovastatin and **IG 2**. In two of the treatment groups, there were a few mice with disproportionally large tumor volumes that skew analyses of aggregated tumor volumes. As an alternative approach, we monitored the growth of individual tumors over time by calculating the ratio of individual tumor volumes at the end of treatment (days 46,47 post-injection) to tumor volumes prior to treatment (day 26 post-injection) (Figure 10C) and of tumor volumes at the end of treatment (days 46,47 post-injection) compared to tumor volumes midway through treatment (days 40,41) (Figure 10D). The box plots presented in Figure 10C,D indicate that combinational treatment significantly reduced S462TY xenograft growth over the course of treatment. Furthermore, given that a ratio of 1 indicates static tumor growth, the box plot in Figure 10D suggests almost no tumor growth during the latter half of combinational treatment.

The in vitro studies suggested that the anti-growth effects of combinational therapy reflected primarily cytostatic activity. Figure 10E shows plots of tumor volumes for 4 individual mice from the combined treatment group prior to treatment, during treatment, and 6 days after terminating treatment. In agreement with the 46,47/40,41 ratio analyses, there was very little, if any, growth between days 41 and 47 in co-treated mice. However, the tumors resumed growth and increased in volume by 35–80% within 6 days of cessation of treatment (Figure 10E).

## 4. Discussion

PAMAM G4 dendrimers have attracted attention as vehicles for intracellular delivery of DNA, shRNA, and numerous chemotherapeutic agents [24]. To our knowledge, this is the first report on the development of prodrug FTI PAMAM G4 dendrimer conjugates. The prodrug *tert*-butylfarnesyl monophosphate FTI (i.e., **3**) required extensive time and vigorous agitation in DMSO in order to dissolve. In contrast, the PAMAM G4 dendrimer conjugate of **3** (i.e., **IG 2**) was soluble in aqueous solutions. While it is possible that conjugation could change the ability of the FTI to interact with its target, **IG 2** retained the potency and efficacy of the non-conjugated prodrug FTI. The toxicity of native PAMAM dendrimers (including generation G4) has been thoroughly documented in a variety of in vitro and in vivo models and is due to the cationic properties of the dendrimer’s terminal amine groups [32,33]. Fortunately, the cytotoxic properties of PAMAM dendrimers can be mitigated by conjugation to neutral or anionic molecules [32,33], which is the strategy we employed. Although dendrimers have attracted considerable attention as a potential mechanism for drug solubilization and delivery [24], their utilization in clinical studies is evolving and has been limited so far [34].

Previous studies indicated that non-cytotoxic/cytostatic concentrations of lovastatin could potentiate the anti-growth properties of prodrug farnesyl monophosphate FTIs like **1** and **2** in NF1 MPNST cell lines [23]. A similar lovastatin-mediated enhancement was observed in the current study, with all of the prodrug *tert*-butylfarnesyl monophosphate FTI dendrimer conjugates examined. In the case of the prodrug FTI **IG 2**, the combinational inhibitory effects on the proliferation of MPNST S462TY and ST88-14 cells and S462TY prenylation in culture were synergistic. Furthermore, combinational lovastatin and **IG 2** treatment, but not single-agent treatment, suppressed the in vivo growth of S462TY sciatic nerve xenografts. Interestingly, the observed combination effects in cultured S462TY cells occurred with concentrations of lovastatin as low as 100 nM and were maximal at 1 μM. Such a range is at the upper level of serum levels (~100 nM) achieved in humans receiving lovastatin (~1 mg/kg/day) for the treatment of hypercholesterolemia [35], and well within the peak serum levels observed in cancer patients receiving 5 mg/kg/day (0.2–1.0 μM) or 10 mg/kg/day (0.5–3.5 μM) for 4 weeks [36]. Both of these latter dosing regimens were well tolerated. Hence, the dosages of lovastatin theoretically needed to potentiate the cytotoxic properties of **IG 2** towards NF1 MPNSTs should be achievable in humans.

The prodrug FTIs examined in our studies presumably suppress farnesylation by competing with endogenous FPP for binding to FTase. We reasoned that co-treatment with doses of lovastatin sufficient to partially suppress the generation of precursors needed for FPP and GGPP synthesis would (1) potentiate FTI effects by reducing the levels of competing endogenous FPP and (2) provide some suppression of GGTase I and GGTase II-mediated geranylgeranylation due to reduced GGPP levels [22]. In addition to the expected results, we also found that higher concentrations of **IG 2** can reduce geranylgeranylation. Specifically, the prenylation of RAP1A and RAB5A, which are exclusively geranylgeranylated, was suppressed in cultures treated with 5 μM of **IG 2** alone (Figure 8A). The mechanism by which **IG 2** might suppress geranylgeranylation is unknown. Although untested, it seems unlikely that **IG 2** suppresses geranylgeranylation by its conversion into a geranylgeranyl moiety. Rather, its inhibitory activity is more likely associated with the FPP backbone of **IG 2**. Indeed, there is remarkable structural conservation in the isoprenoid binding sites of FTase, GGTase I, and GGTase II [37]. Both GGTase I and II are capable of binding FPP and farnesylating select proteins. For example, RhoB exists in vivo as either a farnesylated or geranylgeranylated protein [38]. In vitro studies with recombinant preparations of FTase, GGTase I, and RhoB indicate that GGTase I, but not FTase, was responsible for the farnesylation of RhoB, as well as its geranylgeranylation [39]. Indeed, GGTase I was far better at farnesylating RhoB than it was at geranylgeranylating it. Similarly, Yokoyama et al. [40] reported that GGTase I catalyzes the farnesylation of prenyl acceptors that have a c-terminal leucine in their CAAX box motif. In the case of GGTase-II, in vitro studies indicated that GGTase II can farnesylate Rab7 if the ratio of FPP to GGPP is high [41,42]. Analogous conditions presumably would occur if (1) the concentration of **IG 2** was high relative to endogenous FPP or GGPP levels, and (2) in the combination protocol in which the FTI dendrimer would provide the inhibitory farnesyl co-substrate, the statin would reduce the levels of competing endogenous FPP and GGPP. It should be noted that CAAX box competitive FTIs may not provide significantly greater specificity when it comes to inhibiting prenylation. For example, of the 23 FTIs that did not compete for the isoprenoid binding site on FTase, 17 were identified as potent inhibitors of GGTase-II [43].

The finding that co-treatment with lovastatin and **IG 2** inhibited both farnesylation and geranylgeranylation raises the question of whether the broad effects of the combination treatment on prenylation would limit its usefulness because of generalized toxicity. Preclinical studies have shown that combinations of several FTase and GGTase 1 inhibitors are far more effective than single treatments at inhibiting protein prenylation [44]. When used at concentrations sufficient to suppress the prenylation of HDJ2 (a FTase substrate) and Rap1A (a GGTase I substrate), but not the prenylation of K-Ras, the combinational treatments were well tolerated in several species [44]. However, toxicity became a limiting factor if dosing was sufficient to suppress K-Ras prenylation. In our studies, combinational treatment resulted in a synergistic suppression of proliferation in both S462TY and ST88-14 MPNST cell lines. In contrast, no synergy or additive effects were observed in normal hepatocytes, breast epithelial cells, or Schwann cells. Furthermore, studies in cancer patients receiving dosages of lovastatin sufficient to broadly inhibit prenylation indicated that the treatment was well tolerated and, in some cases, caused the stabilization or remission of the cancer [36]. The basis for why tumor cells would be more responsive is speculative but probably stems from their reliance on processes that provide a growth/survival advantage, in which the function of key proteins requires prenylation.

In the case of NF1 MPNSTs, we know that N-RAS and K-RAS are constitutively activated and potential drivers of proliferation [19,20]. Both RAS isoforms can be either farnesylated or geranylgeranylated, and as such, they are targeted by our combinational approach of using an FTI and lovastatin. The identities of additional driving proteins that require prenylation for transforming activity are speculative. Chemical and genetic approaches for modulating prenylation in *C. elegans* suggested that members of the Rab family (such as Rab5A), which are prenylated by GGTase-II, are critical for cell survival [43]. In particular, the *C. elegans* studies highlighted the importance of those Rabs involved in endocytosis, endosome recycling, and endosome–lysosome fusion/maturation. Rab-dependent processes may be skewed in cancer so as to provide advantages to tumor cells [45]. Rab prenylation may constitute an Achilles’ heel and a potential therapeutic target in NF1 MPNSTs.

## 5. Conclusions

Statins are increasingly being considered for the treatment of a variety of medical conditions, in addition to dyslipidemia. For example, lovastatin and simvastatin have been extensively examined in NF1 patients for their potential abilities to improve synaptic plasticity, behavioral and cognitive functions, as well as phasic alertness [46,47,48,49,50]. Another broad use of statins is in both the mono and combinational treatment of a variety of cancers [51,52,53,54]. A subset of the latter treatments is the combinational use of statins and prenylation inhibitors in the treatment of some cancers [55,56,57]. In the current study, we demonstrate that combinational treatment with lovastatin and an FTI dendrimer suppresses the growth of established NF1 MPNST sciatic nerve xenografted tumors, a Ras-driven tumor type that currently has very limited therapeutic options. Combinational statin and prenylation inhibitor treatment may be relevant to a variety of cancers in which Ras is one of the transforming/neoplastic drivers [58]. For example, in a preclinical study, we found that lovastatin strongly synergized with **IG 2** to suppress the growth of A549 cells, a non-small cell lung carcinoma cell line with mutated K-Ras as a driver (see Supplementary Material Figure S12). The combinatorial approach described in this study could provide a new therapeutic approach to Ras-driven cancers.

## Data Availability

The colony counting data are available on the NF Data Portal (http://www.nf.synapse.org, RRID:SCR_021683) and at the following https://doi.org/10.7303/syn53185180.

## Supplementary Materials

The following supporting information can be downloaded at: https://www.mdpi.com/article/10.3390/cancers16010089/s1, Synthetic procedures; Scheme S1: Scheme for prodrug FTI and prodrug FTI PAMAM G4 dendrimer synthesis; Figures S1–S4 depict original western blots used for composition of Figure 7; Figures S5–S9 depict original western blots used for composition of Figure 8; Figure S10 depicts original western blots used for composition of Figure 9; Figure S11 depicts photographs and MRI analyses of S462TY sciatic nerve xenografts growing in NOD SCID mice; Figure S12 depicts a synergistic inhibition of non-small cell carcinoma A549 colony formation by combinational lovastatin and IG 2 treatment. References [23,28,59,60,61,62,63] are cited in the Supplementary Materials.

## References

[1] Friedman J.M. (1999). Epidemiology of neurofibromatosis type 1. Am. J. Med. Genet..

[2] Arun D., Gutmann D.H. (2004). Recent advances in neurofibromatosis type 1. Curr. Opin. Neurol..

[3] Lynch T.M., Gutmann D.H. (2002). Neurofibromatosis 1. Neurol. Clin..

[4] Perry A., Roth K.A., Banerjee R., Fuller C.E., Gutmann D.H. (2001). NF1 deletions in S-100 protein-positive and negative cells of sporadic and neurofibromatosis 1 (NF1)-associated plexiform neurofibromas and malignant peripheral nerve sheath tumors. Am. J. Pathol..

[5] Ward B.A., Gutmann D.H. (2005). Neurofibromatosis 1: From lab bench to clinic. Pediatr. Neurol..

[6] DeClue J.E., Cohen B.D., Lowy D.R. (1991). Identification and characterization of the neurofibromatosis type 1 protein product. Proc. Natl. Acad. Sci. USA.

[7] Parada L.F. (2000). Neurofibromatosis type 1. Biochim. Biophys. Acta.

[8] Ballester R., Marchuk D., Boguski M., Saulino A., Letcher R., Wigler M., Collins F. (1990). The NF1 locus encodes a protein functionally related to mammalian GAP and yeast IRA proteins. Cell.

[9] Xu G.F., O’Connell P., Viskochil D., Cawthon R., Robertson M., Culver M., Dunn D., Stevens J., Gesteland R., White R. (1990). The neurofibromatosis type 1 gene encodes a protein related to GAP. Cell.

[10] Feldkamp M.M., Angelov L., Guha A. (1999). Neurofibromatosis type 1 peripheral nerve tumors: Aberrant activation of the Ras pathway. Surg. Neurol..

[11] Lau N., Feldkamp M.M., Roncari L., Loehr A.H., Shannon P., Gutmann D.H., Guha A. (2000). Loss of neurofibromin is associated with activation of RAS/MAPK and PI3-K/AKT signaling in a neurofibromatosis 1 astrocytoma. J. Neuropathol. Exp. Neurol..

[12] Hiatt K.K., Ingram D.A., Zhang Y., Bollag G., Clapp D.W. (2001). Neurofibromin GTPase-activating protein-related domains restore normal growth in Nf1^−/−^ cells. J. Biol. Chem..

[13] Basso A.D., Kirschmeier P., Bishop W.R. (2006). Lipid posttranslational modifications. Farnesyl transferase inhibitors. J. Lipid Res..

[14] Yan N., Ricca C., Fletcher J., Glover T., Seizinger B.R., Manne V. (1995). Farnesyltransferase inhibitors block the neurofibromatosis type I (NF1) malignant phenotype. Cancer Res..

[15] Kim H.A., Ling B., Ratner N. (1997). Nf1-deficient mouse Schwann cells are angiogenic and invasive and can be induced to hyperproliferate: Reversion of some phenotypes by an inhibitor of farnesyl protein transferase. Mol. Cell Biol..

[16] Widemann B.C., Dombi E., Gillespie A., Wolters P.L., Belasco J., Goldman S., Korf B.R., Solomon J., Martin S., Salzer W. (2014). Phase 2 randomized, flexible crossover, double-blinded, placebo-controlled trial of the farnesyltransferase inhibitor tipifarnib in children and young adults with neurofibromatosis type 1 and progressive plexiform neurofibromas. Neuro Oncol..

[17] Lerner E.C., Zhang T.T., Knowles D.B., Qian Y., Hamilton A.D., Sebti S.M. (1997). Inhibition of the prenylation of K-Ras, but not H- or N-Ras, is highly resistant to CAAX peptidomimetics and requires both a farnesyltransferase and a geranylgeranyltransferase I inhibitor in human tumor cell lines. Oncogene.

[18] Whyte D.B., Kirschmeier P., Hockenberry T.N., Nunez-Oliva I., James L., Catino J.J., Bishop W.R., Pai J.K. (1997). K- and N-Ras are geranylgeranylated in cells treated with farnesyl protein transferase inhibitors. J. Biol. Chem..

[19] Mattingly R.R., Kraniak J.M., Dilworth J.T., Mathieu P., Bealmear B., Nowak J.E., Benjamins J.A., Tainsky M.A., Reiners J.J. (2006). The mitogen-activated protein kinase/extracellular signal-regulated kinase kinase inhibitor PD184352 (CI-1040) selectively induces apoptosis in malignant schwannoma cell lines. J. Pharmacol. Exp. Ther..

[20] Brossier N.M., Prechtl A.M., Longo J.F., Barnes S., Wilson L.S., Byer S.J., Brosius S.N., Carroll S.L. (2015). Classic Ras Proteins Promote Proliferation and Survival via Distinct Phosphoproteome Alterations in Neurofibromin-Null Malignant Peripheral Nerve Sheath Tumor Cells. J. Neuropathol. Exp. Neurol..

[21] Mattingly R.R., Gibbs R.A., Menard R.E., Reiners J.J. (2002). Potent suppression of proliferation of a10 vascular smooth muscle cells by combined treatment with lovastatin and 3-allylfarnesol, an inhibitor of protein farnesyltransferase. J. Pharmacol. Exp. Ther..

[22] Wojtkowiak J.W., Gibbs R.A., Mattingly R.R. (2009). Working together: Farnesyl transferase inhibitors and statins block protein prenylation. Mol. Cell Pharmacol..

[23] Wojtkowiak J.W., Fouad F., LaLonde D.T., Kleinman M.D., Gibbs R.A., Reiners J.J., Borch R.F., Mattingly R.R. (2008). Induction of apoptosis in neurofibromatosis type 1 malignant peripheral nerve sheath tumor cell lines by a combination of novel farnesyl transferase inhibitors and lovastatin. J. Pharmacol. Exp. Ther..

[24] Jedrych M., Borowska K., Galus R., Jodlowska-Jedrych B. (2014). The evaluation of the biomedical effectiveness of poly(amido)amine dendrimers generation 4.0 as a drug and as drug carriers: A systematic review and meta-analysis. Int. J. Pharm..

[25] Guo M., Joiakim A., Dudley D.T., Reiners J.J. (2001). Suppression of 2,3,7,8-tetrachlorodibenzo-p-dioxin (TCDD)-mediated CYP1A1 and CYP1B1 induction by 12-O-tetradecanoylphorbol-13-acetate: Role of transforming growth factor beta and mitogen-activated protein kinases. Biochem. Pharmacol..

[26] Jackson N.M., Kocarek T.A. (2008). Suppression of CYP2B induction by alendronate-mediated farnesyl diphosphate synthase inhibition in primary cultured rat hepatocytes. Drug Metab. Dispos..

[27] Mosmann T. (1983). Rapid colorimetric assay for cellular growth and survival: Application to proliferation and cytotoxicity assays. J. Immunol. Methods.

[28] Clark M.K., Scott S.A., Wojtkowiak J., Chirco R., Mathieu P., Reiners J.J., Mattingly R.R., Borch R.F., Gibbs R.A. (2007). Synthesis, biochemical, and cellular evaluation of farnesyl monophosphate prodrugs as farnesyltransferase inhibitors. J. Med. Chem..

[29] Wojtkowiak J.W., Sane K.M., Kleinman M., Sloane B.F., Reiners J.J., Mattingly R.R. (2011). Aborted autophagy and nonapoptotic death induced by farnesyl transferase inhibitor and lovastatin. J. Pharmacol. Exp. Ther..

[30] Duarte D., Vale N. (2022). Evaluation of synergism in drug combinations and reference models for future orientations in oncology. Curr. Res. Pharmacol. Drug Discov..

[31] Maurer-Stroh S., Koranda M., Benetka W., Schneider G., Sirota F.L., Eisenhaber F. (2007). Towards complete sets of farnesylated and geranylgeranylated proteins. PLoS Comput. Biol..

[32] Li X., Naeem A., Xiao S., Hu L., Zhang J., Zheng Q. (2022). Safety Challenges and Application Strategies for the Use of Dendrimers in Medicine. Pharmaceutics.

[33] Janaszewska A., Lazniewska J., Trzepinski P., Marcinkowska M., Klajnert-Maculewicz B. (2019). Cytotoxicity of Dendrimers. Biomolecules.

[34] Kisakova L.A., Apartsin E.K., Nizolenko L.F., Karpenko L.I. (2023). Dendrimer-Mediated Delivery of DNA and RNA Vaccines. Pharmaceutics.

[35] Pan H.Y., DeVault A.R., Wang-Iverson D., Ivashkiv E., Swanson B.N., Sugerman A.A. (1990). Comparative pharmacokinetics and pharmacodynamics of pravastatin and lovastatin. J. Clin. Pharmacol..

[36] Chan K.K., Oza A.M., Siu L.L. (2003). The statins as anticancer agents. Clin. Cancer Res..

[37] Zhang H., Seabra M.C., Deisenhofer J. (2000). Crystal structure of Rab geranylgeranyltransferase at 2.0 A resolution. Structure.

[38] Adamson P., Marshall C.J., Hall A., Tilbrook P.A. (1992). Post-translational modifications of p21rho proteins. J. Biol. Chem..

[39] Armstrong S.A., Hannah V.C., Goldstein J.L., Brown M.S. (1995). CAAX geranylgeranyl transferase transfers farnesyl as efficiently as geranylgeranyl to RhoB. J. Biol. Chem..

[40] Yokoyama K., McGeady P., Gelb M.H. (1995). Mammalian protein geranylgeranyltransferase-I: Substrate specificity, kinetic mechanism, metal requirements, and affinity labeling. Biochemistry.

[41] Owen D.J., Alexandrov K., Rostkova E., Scheidig A.J., Goody R.S., Waldmann H. (1999). Chemo-Enzymatic Synthesis of Fluorescent Rab 7 Proteins: Tools to Study Vesicular Trafficking in Cells. Angew. Chem. Int. Ed..

[42] Thoma N.H., Iakovenko A., Owen D., Scheidig A.S., Waldmann H., Goody R.S., Alexandrov K. (2000). Phosphoisoprenoid binding specificity of geranylgeranyltransferase type II. Biochemistry.

[43] Lackner M.R., Kindt R.M., Carroll P.M., Brown K., Cancilla M.R., Chen C., de Silva H., Franke Y., Guan B., Heuer T. (2005). Chemical genetics identifies Rab geranylgeranyl transferase as an apoptotic target of farnesyl transferase inhibitors. Cancer Cell.

[44] Lobell R.B., Omer C.A., Abrams M.T., Bhimnathwala H.G., Brucker M.J., Buser C.A., Davide J.P., deSolms S.J., Dinsmore C.J., Ellis-Hutchings M.S. (2001). Evaluation of farnesyl:protein transferase and geranylgeranyl:protein transferase inhibitor combinations in preclinical models. Cancer Res..

[45] Mellman I., Yarden Y. (2013). Endocytosis and cancer. Cold Spring Harb. Perspect. Biol..

[46] Jung N.H., Egert-Schwender S., Schossow B., Kehl V., Wahllander U., Brich L., Janke V., Blankenstein C., Zenker M., Mall V. (2023). Improvement of synaptic plasticity and cognitive function in RASopathies-a monocentre, randomized, double-blind, parallel-group, placebo-controlled, cross-over clinical trial (SynCoRAS). Trials.

[47] Ullrich N.J., Payne J.M., Walsh K.S., Cutter G., Packer R., North K., Rey-Casserly C., Consortium N.F.C.T. (2020). Visual spatial learning outcomes for clinical trials in neurofibromatosis type 1. Ann. Clin. Transl. Neurol..

[48] Payne J.M., Hearps S.J.C., Walsh K.S., Paltin I., Barton B., Ullrich N.J., Haebich K.M., Coghill D., Gioia G.A., Cantor A. (2019). Reproducibility of cognitive endpoints in clinical trials: Lessons from neurofibromatosis type 1. Ann. Clin. Transl. Neurol..

[49] van der Vaart T., Rietman A.B., Plasschaert E., Legius E., Elgersma Y., Moll H.A., Group N.S.S. (2016). Behavioral and cognitive outcomes for clinical trials in children with neurofibromatosis type 1. Neurology.

[50] Mainberger F., Jung N.H., Zenker M., Wahllander U., Freudenberg L., Langer S., Berweck S., Winkler T., Straube A., Heinen F. (2013). Lovastatin improves impaired synaptic plasticity and phasic alertness in patients with neurofibromatosis type 1. BMC Neurol..

[51] Tilija Pun N., Jeong C.-H. (2021). Statin as a Potential Chemotherapeutic Agent: Current Updates as a Monotherapy, Combination Therapy, and Treatment for Anti-Cancer Drug Resistance. Pharmaceuticals.

[52] Xia L., Ding S., Wang X., Zhang X., Zhu L., Zhang H., Li H. (2022). Advances in ovarian cancer treatment using a combination of statins with other drugs. Front. Pharmacol..

[53] Issat T., Nowis D., Bil J., Winiarska M., Jakobisiak M., Golab J. (2011). Antitumor effects of the combination of cholesterol reducing drugs. Oncol. Rep..

[54] Zaky M.Y., Fan C., Zhang H., Sun X.F. (2023). Unraveling the Anticancer Potential of Statins: Mechanisms and Clinical Significance. Cancers.

[55] El-Refai S.M., Brown J.D., Arnold S.M., Black E.P., Leggas M., Talbert J.C. (2017). Epidemiologic Analysis Along the Mevalonate Pathway Reveals Improved Cancer Survival in Patients Who Receive Statins Alone and in Combination with Bisphosphonates. JCO Clin. Cancer Inform..

[56] Garcia-Ruiz C., Morales A., Fernandez-Checa J.C. (2012). Statins and protein prenylation in cancer cell biology and therapy. Anticancer Agents Med. Chem..

[57] Sarrabayrouse G., Pich C., Teiti I., Tilkin-Mariame A.F. (2017). Regulatory properties of statins and rho gtpases prenylation inhibitiors to stimulate melanoma immunogenicity and promote anti-melanoma immune response. Int. J. Cancer.

[58] Brock E.J., Ji K., Reiners J.J., Mattingly R.R. (2016). How to Target Activated Ras Proteins: Direct Inhibition vs. Induced Mislocalization. Mini Rev. Med. Chem..

[59] Müller T.E., Pleier A.K. (1999). Intramolecular hydroamination of alkynes catalysed by late transition metals. J. Chem. Soc. Dalton Trans..

[60] Lu B., Li C., Zhang L. (2010). Gold-Catalyzed Highly Regioselective Oxidation of C−C Triple Bonds without Acid Additives: Propargyl Moieties as Masked α,β-Unsaturated Carbonyls. J. Am. Chem. Soc..

[61] Knor S., Modlinger A., Poethko T., Schottelius M., Wester H.J., Kessler H. (2007). Synthesis of novel 1,4,7,10-tetraazacyclodecane-1,4,7,10-tetraacetic acid (DOTA) derivatives for chemoselective attachment to unprotected polyfunctionalized compounds. Chemistry.

[62] Theodossiou T.A., Pantos A., Tsogas I., Paleos C.M. (2008). Guanidinylated dendritic molecular transporters: Prospective drug delivery systems and application in cell transfection. ChemMedChem.

[63] Feichtinger K., Zapf C., Sings H.L., Goodman M. (1998). Diprotected Triflylguanidines:  A New Class of Guanidinylation Reagents. J. Org. Chem..

