# Supplementary material for: Synergistic Suppression of NF1 Malignant Peripheral Nerve Sheath Tumor Cell Growth in Culture and Orthotopic Xenografts by Combinational Treatment with Statin and Prodrug Farnesyltransferase Inhibitor PAMAM G4 Dendrimers
Source: Cancers (Basel). 2023 Dec 23;16(1):89. doi: 10.3390/cancers16010089 (PMC10778372; doi:10.3390/cancers16010089)
Supplement: Supplementary file 1 [file cancers-16-00089-s001.zip › cancers-2748955_Supplementary Synthetic Procedures_proof_final.pdf]

Synthetic Procedures

Synthesis of tert-butylfarnesyl monophosphate prodrug FTIs

Synthesis of Compounds **1**, **2**, **3**, **4**, and **5**.

The syntheses of farnesyl monophosphate prodrugs FTI-1 and FTI-2 (labeled as **1** and **2**, respectively; see Figure 1 in the main manuscript) have been described [1,2]. Compound **3** (also shown in Figure 1 in the main manuscript) was synthesized using a similar strategy, which is outlined in Scheme S1. 5-cyano-1-pentyne (Acros Organics via Fisher Scientific, Pittsburgh, PA) was converted to 6-amino-1-hexyne, **(4,** Scheme S1), as previously described [3], followed by conversion to the Boc derivative **5** (Scheme S1) [4].

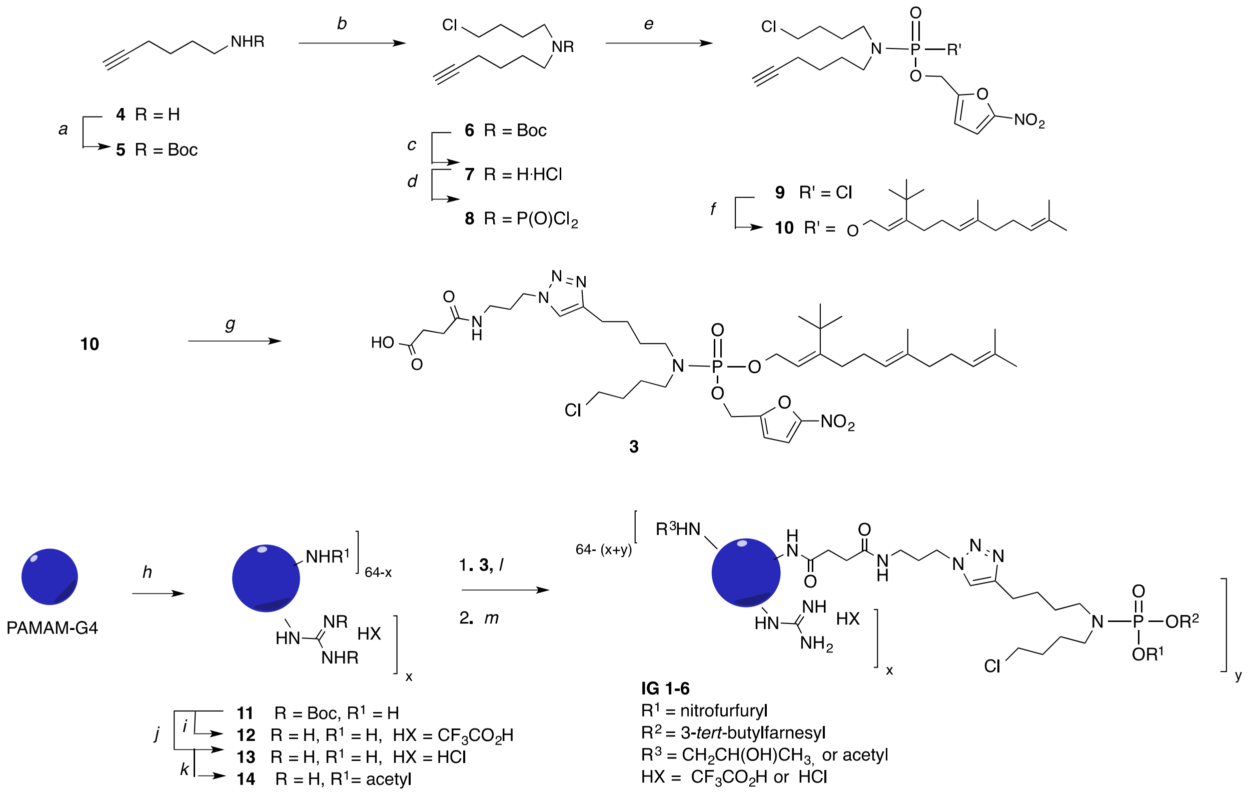

(*a*) DIEA, Boc-anhydride, DCM, 4°C to rt; (*b*) 1. NaH, DMF, 0°C, 20 min 2. Br $(CH_2)_4Cl$; (*c*) EtOAc, HCl; (*d*) $POCl_3$, DCM, 4°C, DIEA; (*e*) Nitrofurfuryl alcohol, LiHMDS, THF, -78°C; (*f*) *tert*-butylfarnesol, DCM, NEt_3,, $TiCl_4$, 4°C; (*g*) 3-(3-azidopropylcarbamoyl)propanoic acid, $CuSO_4 \cdot 5\,H_2O$, sodium ascorbate, tBuOH/H_2O; (*h*) $(BocNH)_2C$=NTf, MeOH; (*i*) TFA, DCM; (*j*) HCl, MeOH/H_2O; (*k*) 1. Ac_2O, NEt_3 2. 6M HCl/MeOH; (*l*) **12**, HOBT, DIEA, DMF, HATU or **13**, EDC· HCl; (*m*) propylene oxide or Ac_2O, NEt_3, MeOH

Scheme S1. Scheme for prodrug FTI and prodrug FTI PAMAM G4 dendrimer synthesis.

Synthesis of Compound **6**

NaH, 60% dispersion in mineral oil (2.78 g, 72.54 mmol, 1.8 equiv) in DMF (10 mL) was cooled to 4°C. Compound **5** (Scheme S1; 7.95 g, 40.30 mmol) in DMF (10 mL) was added to the NaH dispersion. After 35 min at 4°C 1-bromo-4-chlorobutane (7.9 mL, 68.65 mmol, 1.7 equiv) was added and the reaction came to rt gradually. The reaction mixture was poured into pentane/water (1/1, 100 mL) and the aqueous phase was extracted with pentane (3 x 75 mL). The combined extracts were washed with brine, dried over Na_2SO_4,

and concentrated to a pale yellow oil. The crude product was purified by flash column chromatography (gradient elution with 0%, 2%, 4%, 6% acetone/hexanes) to provide the product as a clear oil (10.33 g, 89%).^{1}H NMR ($CDCl_3$): δ 3.55 (t, 2H); 3.20 (bm, 4H); 2.20 (dt. 2H); 1.94 (t, 1H); 1.75-1.50 (m, 8H); 1.45 (s, 9H). HRMS ($C_{15}H_{26}ClNO_2$) calcd 288.1730, found 288.1729.

Synthesis of Compound **7**

Compound **6** (Scheme S1; 10.33 g, 35.88 mmol) in 100 mL EtOAc was treated with HCl gas for 45 min. The reaction mixture was capped and stirred at rt overnight. The white precipitate that formed was filtered, rinsed with Et_2O and dried under vacuum (7.46 g, 93%). ^{1}H NMR (D_2O): δ 3.54 (m, 2H); 2.97 (m, 4H); 2.27 (bs, 1H); 2.17 (m, 2H); 1.75-1.67 (m, 6H); 1.49 (m, 2H). HRMS ($C_{10}H_{18}ClN$) calcd 188.1206, found 188.1203.

Synthesis of Compound **8**

Compound **7** (Scheme S1; 0.68 g, 3.05 mmol) in DCM (10 mL) was cooled to 4°C and was treated with $POCl_3$ (0.34 mL, 3.66 mmol, 1.2 equiv). Diisopropylethylamine (1.2 mL, 7.02 mmol, 2.3 equiv) was added and the reaction came gradually to rt. After 18h the reaction mixture was quenched with saturated NH_4Cl solution. The phases were separated and the organic phase was dried over Na_2SO_4 and concentrated to an amber oil. The crude product was purified by flash column chromatography (gradient elution 0 to 15% Et_2O/hexanes) (0.72 g, 78%). ^{1}H NMR ($CDCl_3$): δ 3.56 (t, 2H); 3.30-3.20 (m, 4H); 2.25 (dt. 2H); 1.97 (t, 1H); 1.97-1.71 (m, 6H); 1.55 (m, 2H). ^{31}P NMR ($CDCl_3$): δ-7.60 (s). HRMS ($C_{10}H_{17}Cl_3NOP$) calcd 304.0192, found 304.0193.

Synthesis of Compound **9**

Nitrofurfuryl alcohol (0.72 g, 2.36 mmol, 1.05 equiv) in THF (10 mL) was cooled to -78°C. LiHMDS (1M/THF, 2.6 mL, 2.60 mmol, 1.1 equiv) was added by syringe. A solution of Compound **8** (Scheme S1; 0.72 g, 2.36 mmol) in THF (10 mL) was prepared, cooled to -78°C, and was then treated with the alkoxide solution. After 3h at -78°C the reaction mixture was quenched with saturated NH_4Cl solution and after warming to rt was extracted with EtOAc (3 x 25 mL). The combined organic extracts were dried over Na_2SO_4 and were concentrated to a dark amber oil. Purification by flash column chromatography (gradient elution 0%, 10%, 20%, 30%, 40% Et_2O/hexanes) provided the product as a pale yellow oil (0.56 g, 57%). ^{1}H NMR ($CDCl_3$): δ 7.27 (d, 1H); 6.71 (d, 1H); 5.13 (m, 2H); 3.52 (t, 2H); 3.18-3.01 (m, 4H); 2.19 (dt. 2H); 1.97 1.93 (t, 1H); 1.77-1.65 (m, 6H); 1.49 (m, 2H). ^{31}P NMR ($CDCl_3$): δ-8.20 (s). HRMS ($C_{15}H_{21}Cl_2N_2O_5P$) calcd (M + Na) 433.0463, found 433.0468.

Synthesis of Compound **10**

Tert-butylfarnesol [2] (0.28 g, 1.05 mmol) dissolved in DCM (2 mL) was added to Compound **9** (Scheme S1; 0.43 g, 1.05 mmol) in DCM (11 mL). The mixture was cooled to 4° C and NEt_3 (0.18 mL, 1.26 mmol, 1.2 equiv) and 1M $TiCl_4$ in CH_2Cl_2 (0.21 mL, 0.21 mmol, 0.2 equiv) were added. The reaction was capped and kept in the refrigerator for 2 days. Solvent was removed and the residue was applied to a silica gel column and was eluted first with 20% hexanes/DCM, then with 3% Et_2O/DCM to isolate the product as a yellow oil (0.35 g, 52%). ^{1}H NMR ($CDCl_3$): δ 7.26 (d, 1H); 6.64 (d, 1H); 5.21 (t, 1H); 5.16 (m, 2H); 4.98 (d, 2H); 4.72 (m, 2H); 3.55 (t, 2H); 3.07-3.01 (m, 4H); 2.20 (t. 2H); 2.05 (bs, 1H); 1.99-1.94 (m, 8H); 1.76-1.48 (m, 8H); 1.67 (s, 3H); 1.60 (s, 6H); 1.12 (s, 9H). ^{31}P NMR ($CDCl_3$): δ-15.23 (s). HRMS ($C_{33}H_{52}ClN_2O_6P$) calcd $(M + Na)^+$ 661.3149, found 661.5154.

Synthesis of Compound **3**

Compound **10** (Scheme S1; 0.35 g, 0.55 mmol) in *tert*-butanol (5.6 mL) was treated with 3-(3-azidopropylcarbamoyl)propanoic acid [5] (0.11 g, 0.55 mmol, 1.0 equiv). 1.8 mL of 0.08M $CuSO_4$ was added

to the azide and alkyne followed by sodium ascorbate (0.04 g, 0.22 mmol, 0.4 equiv). The reaction was capped and stirred at rt overnight. Organic solvent was removed by rotovap, 5 mL of EtOAc was added, and the reaction mixture was filtered through celite. The filtrate was diluted with 5 mL of H_2O and was extracted with EtOAc (3 x 10 mL). Combined extracts were washed with brine, dried over Na_2SO_4 and concentrated to a gold-colored syrup. Purification by column chromatography with 10%Et_2O/DCM followed by 4% MeOH/DCM provided the product as a yellow oil (0.36 g, 76%). ^1H NMR ($CDCl_3$): δ 7.48 (s, 1H); 7.25 (d, 1H); 6.64 (d, 1H); 5.15-5.06 (m, 3H); 4.96 (d, 2H); 4.68 (m, 2H); 4.36 (t, 2H); 3.51 (t, 2H); 3.22 (m, 2H); 3.06-2.96m, 4H); 2.67 (m, 4H); 2.45 (t. 2H); 2.06-1.95 (m, 10H); 1.74-1.52 (m, 8H); 1.65 (s, 3H); 1.57 (s, 6H); 1.09 (s, 9H). ^{31}P NMR ($CDCl_3$): δ-14.77 (s). HRMS ($C_{41}H_{67}ClN_5O_{11}P$) calcd 872.4342, found 872.4358.

Synthesis of prodrug FTI dendrimers

To increase the hydrophilicity of the tert-butylfarnesyl monophosphate prodrugs we conjugated them to PAMAM G4, which contains 64 surface primary amino groups. A generalized dendrimer-prodrug conjugate is outlined in Figure 1 in the main manuscript. Guanidinium groups were incorporated to enhance hydrophilicity and cellular permeability [6]. Prodrugs were connected to the dendrimer via a linker, and unmodified surface amines were capped to minimize cation-induced cytotoxicity. Optimization of guanidinium groups (x), prodrug moieties (y), and capped surface amines (z) was explored through synthesis of various analogs as outlined in Scheme S1.

The number of guanidinium residues per dendrimer-prodrug conjugate was reliably determined using ^1H NMR integration values of the Boc protons of the protected guanidinylated dendrimers 11 (Scheme S1). However, determination of y, the number of prodrug residues per dendrimer (see Figure 1 in the main manuscript), by ^1H NMR integration was less reliable since the corresponding ^1H NMR spectra are complicated and resonances are broad and overlapping. For this reason UV analysis was used to assess prodrug loading of the dendrimer-prodrug conjugates. The extinction coefficient of 3 was measured at 310 nm, and the UV absorbance at 310 nm of the dendrimer-prodrug conjugates in solutions of defined concentrations were used according to the Beer-Lambert Law to determine prodrug loading as μmol/mg conjugate.

Synthesis of Compound **11**

PAMAM-G4 as a 10% solution in MeOH (2 mL, 0.2 g, 0.014 mmol dendrimer, 0.90 mmol NH_2 groups) was treated with N,N′-di-Boc-N′′-triflylguanidine (0.09 g, 0.22 mmol) prepared as described by Feichtinger et al. [7]. The reaction mixture was capped and stirred at rt for 18h. Solvent was removed by rotovap and the resulting white solid was coevaporated with DCM (3 x 2mL). A small portion of the crude was purified by dialysis for analysis and the remainder was used without further purification (0.24 g, 93%). The number of di-Boc guanidine groups was assessed to be 17 by integration of the^1H NMR. ^1H NMR (D_2O): δ 3.24 (248H); 3.06-2.73 (m, 376H); 2.54 (m, 123H); 2.33 (m, 248H); 1.39 (s, 155H); 1.471.33 (s, 155H).

It should be noted that the number of guanidine moieties per PAMAM-G4 dendrimer could be varied by altering the concentration of N,N′-di-Boc-N′′-triflylguanidine, and different batches of compound **11**, (Scheme S1) with varied numbers of guanidine moieties, were used in the synthesis of prodrug FTI PAMAM-G4 conjugated dendrimers.

Synthesis of Compound **12**

Compound **11** (Scheme S1; 0.15 g, 0.007 mmol) was treated with 25% TFA/$CHCl_3$. The suspension was capped and stirred at rt for 2 days. Solvent was removed and the residue was triturated with CH_2Cl_2 (3 x 2

mL). Purification by dialysis against pure H_2O provided the product as a white solid (0.12 g, 57%). ^{1}H NMR (D_2O): δ 3.40 (m, 64H); 3.24 (m, 250H); 3.05 (m, 64H); 2.80 (m, 248H); 2.61(m, 124H); 2.37 (s, 248H).

Synthesis of Compound **13**

Compound **11** (Scheme S1; 1.25 g, 0.069 mmol) in 10 mL MeOH was treated with 10 mL 6M HCl/MeOH (1/1). The solution was capped and stirred at rt overnight. Solvents were removed under reduced pressure and the residue was co-evaporated with CH_3CN (3 x 10 mL). The remaining solid was triturated with DCM (3 x 5 mL) and the material was dried under vacuum to give a white foam (0.83 g, 62%). This material was used without further purification; however, a small portion of the product was further purified by dialysis against pure water to provide a sample for analysis.^{1}H NMR (D_2O): δ 3.44 (m, 245H); 3.30-2.93 (m, 500H); 2.60 (m, 248H).

Synthesis of Compound **14** (aka **IG 6**)

Compound **13** (x = 16) (Scheme S1; 0.05 g, 3.2 μmol) in 2 mL MeOH was treated with 0.2 mL Ac_2O and 0.3 mL NEt_3. The solution was capped and stirred at rt overnight. Solvents were removed under reduced pressure and the residue was taken up in 1 mL H_2O and was dialyzed against pure water. After three water changes, the dialysis mixture was acidified with 0.5 mL 6M HCl/MeOH, and the resulting mixture was passed through a column of sephadex LH-20 with MeOH as eluent. Fractions containing only baseline spots on thin-layer chromatography when eluted with 5% MeOH/EtOAc were collected, concentrated, taken up in 3 mL H_2O and lyophilized to give a white foam (0.04 g, 71%). ^{1}H NMR (MeOH-d_4): δ 3.90-3.20 (m, 993H); 2.90 (m, 248H); 2.09 (s, 138H).

General procedure for the synthesis of dendrimer prodrug conjugates by conjugation of Compounds ***12*** *and* ***3*** *using HOBt/HATU to yield Compounds* ***IG 1***, ***IG 3***, ***IG 5***

Compound **12** (Scheme S1; 0.20 g, 0.0070 mmol dendrimer, 0.17 mmol NH_2 groups) was dissolved in 0.6 mL dry DMF. In a separate vial, compound **3** (0.22 g, 0.26 mmol) and HOBt (0.04 g, 0.29 mmol, 1.1 equiv) in 0.4 mL dry DMF were treated with HATU (0.11 g, 0.29 mmol, 1.1 equiv). The reaction vial was swirled for 45 s and the contents were added to the dendrimer solution. The mixture was stirred under Ar overnight. As much DMF as possible was removed under reduced pressure and the resulting sticky oil was taken up in MeOH (2 mL) and propylene oxide (1 mL) was added. The vial was capped and stirred at rt overnight. The reaction mixture was concentrated under reduced pressure and the residue was passed through a column of sephadex LH-20 with MeOH/DCM (1/1) as eluent. Fractions containing only baseline spots on thin-layer chromatography when eluted with 5% MeOH/EtOAc were collected and concentrated to yield 0.22g of material which still showed some HOBt/HATU by-products by ^{1}H NMR. Repeated sephadex chromatography was required to provide the pure CF_3CO_2H salts. To obtain acetate salts, the partially-purified CF_3CO_2H salts were dissolved in H_2O, shaken with Dowex 1-X10 anion exchange resin, and washed extensively with 1N HCl and then 1N HOAc. After 30 min the resin was filtered and washed with H_2O (2 x 5 mL). The filtrate was lyophilized to provide the acetate salt as an amber-colored foam of 0.132 g.

Integration values for the compounds below are based on setting the prodrug nitrofurfuryl vinyl protons at 1.

IG 1. ^{1}H NMR (MeOH-d_4): δ 7.78 (s, 1H); 7.44 (d, 1H); 6.82 (d, 1H); 5.23-5.02 (m, 5H); 4.74 (t, 1H); 4.40 (t, 1H); 3.57 (t, 1H); 3.19 (bs, 3H); 2.89-2.60 (m, 9H), 2.49-2.39 (m, 14 H); 2.06-1.93 (m, 10H); 1.71-1.47 (m, 22H); 1.13 (s, 9H). ^{31}P NMR (MeOH-d_4): δ -13.94 (s).

IG 3. ^{1}H NMR (MeOH-d_4): δ 7.77 (s, 1H); 7.44 (d, 1H); 6.82 (d, 1H); 5.23-5.02 (m, 5H); 4.74 (t, 1H); 4.40 (t, 1H); 3.56 (t, 1H); 3.07 (bs, 3H); 2.78-2. (m, 9H), 2.57-2.36 (m, 13H); 2.05-1.95 (m, 10H); 1.65-1.58 (m, 15H); 1.13 (s, 9H). ^{31}P NMR (MeOH-d_4): δ -13.71 (s).

IG 5. ^{1}H NMR (MeOH-d_4): δ 7.76 (s, 1H); 7.43 (d, 1H); 6.80 (d, 1H); 5.23-4.92 (m, 5H); 4.73 (t, 1H); 4.39 (t, 1H); 3.55 (t, 1H); 3.29-2.91 (m, 6 H), 2.69-2.61 (m, 4 H); 2.61-2.48 (m, 12H); 2.05-1.95 (m, 8H); 1.68-1.58 (m, 22H); 1.13 (s, 9H). ^{31}P NMR (MeOH-d_4): δ -14.89 (s).

*General procedure for the synthesis of dendrimer prodrug conjugates by conjugation of Compounds **13** and **3** using EDC·HCl to generate Compounds **IG 2**, **IG 4***

Compound **13** (Scheme S1; 0.12 g, 0.0063 mmol dendrimer, 0.29 mmol NH_2 groups) was dissolved in H_2O (0.75 mL). In a separate vial compound **3** (Scheme S1; 0.16 g, 0.182 mmol) was dissolved in acetone (1 mL) to make a cloudy solution that was added to the dendrimer. To this mixture was added EDC·HCl (0.04 g, 0.22 mmol, 1.2 equivalent) and DIEA (0.07 mL, 0.41 mmol, 2.3 equiv). The cloudy solution was capped and stirred at rt overnight. Solvents were removed under reduced pressure and the residue was taken up in MeOH (1.5 mL) and was treated with Ac_2O (0.1 mL) and NEt_3 (0.3 mL). After 20 h solvent was removed, the residue was taken up in H_2O (10 mL) and the solution was purified by dialysis against pure H_2O. The product was obtained as an amber-colored foam, 0.07 g.

Integration values for the compounds below are based on setting the prodrug nitrofurfuryl vinyl protons at 1.

IG 2. ^{1}H NMR (MeOH-d_4): δ 7.78 (s, 1H); 7.43 (d, 1H); 6.81 (d, 1H); 5.23-5.02 (m, 5H); 4.75 (t, 1H); 4.40 (t, 1H); 3.56 (t, 1H); 3.19 (bs, 3H); 2.87-2.70 (m, 10H), 2.70-2.68 (m, 7H); 2.52-2.43 (m, 20H); 2.06-1.95 (m, 13H); 1.74-1.59 (m, 17H); 1.20 (s, 9H). ^{31}P NMR (MeOH-d_4): δ -13.68 (s).

IG 4. ^{1}H NMR (MeOH-d_4): δ 7.77 (s, 1H); 7.44 (d, 1H); 6.81 (d, 1H); 5.23-5.01 (m, 5H); 4.76 (t, 1H); 4.39 (t, 1H); 3.57 (t, 1H); 3.21 (bs, 4H); 2.86-2.74 (m, 12H), 2.59-2.43 (m, 32H); 2.06-1.95 (m, 26H); 1.74-1.53 (m, 17H); 1.13 (s, 9H). ^{31}P NMR (MeOH-d_4): δ -14.37 (s).

*General procedure for synthesis of dendrimer-prodrug conjugates by conjugation of Compounds **13** and **3** using 1,1′-carbonyldiimidazole (CDI)*

Compound **13** (Scheme S1; 0.47 g, 0.024 mmol dendrimer, 1.16 mmol NH_2 groups) was dissolved in H_2O (9 mL). Imidazole was added to bring the pH to 7. In a separate vial, compound **3** (0.43 g, 0.51 mmol) was dissolved in acetone (9 mL) and was treated with CDI (0.20 g, 1.23 mmol, 2.4 equivalent). A small aliquot was removed and concentrated after 40 min. Examination by ^{1}H NMR showed a complete downfield shift of the methylene next to the carboxylic acid group in compound **3**. Thus, the activated acid was added to the dendrimer and the cloudy mixture was capped and stirred at rt overnight. Ac_2O (0.55 mL) was added to the mixture. After 5 min the pH was found to be approximately 5 and imidazole was added to bring the pH to 7. After stirring at rt for 20 h, the acetone was removed and the cloudy aqueous solution was washed multiple times with Et_2O to remove the bulk of unreacted Ac_2O and imidazole. The remaining material was passed through a sephadex LH-20 column with MeOH as eluent to provide the product as a brownish-yellow colored foam, 0.40 g.

References

1. Wojtkowiak, J.W.; Fouad, F.; LaLonde, D.T.; Kleinman, M.D.; Gibbs, R.A.; Reiners, J.J., Jr.; Borch, R.F.; Mattingly, R.R. Induction of apoptosis in neurofibromatosis type 1 malignant peripheral nerve sheath tumor cell lines by a combination of novel farnesyl transferase inhibitors and lovastatin. *J Pharmacol Exp Ther* **2008**, *326*, 1-11, doi:10.1124/jpet.107.135830.

2. Clark, M.K.; Scott, S.A.; Wojtkowiak, J.; Chirco, R.; Mathieu, P.; Reiners, J.J., Jr.; Mattingly, R.R.; Borch, R.F.; Gibbs, R.A. Synthesis, biochemical, and cellular evaluation of farnesyl monophosphate prodrugs as farnesyltransferase inhibitors. *J Med Chem* **2007**, *50*, 3274-3282, doi:10.1021/jm0701829.
3. E. Müller, T.; Pleier, A.-K. Intramolecular hydroamination of alkynes catalysed by late transition metals. *Journal of the Chemical Society, Dalton Transactions* **1999**, 583-588, doi:10.1039/A808938H.
4. Lu, B.; Li, C.; Zhang, L. Gold-Catalyzed Highly Regioselective Oxidation of C−C Triple Bonds without Acid Additives: Propargyl Moieties as Masked α,β-Unsaturated Carbonyls. *Journal of the American Chemical Society* **2010**, *132*, 14070-14072, doi:10.1021/ja1072614.
5. Knor, S.; Modlinger, A.; Poethko, T.; Schottelius, M.; Wester, H.J.; Kessler, H. Synthesis of novel 1,4,7,10-tetraazacyclodecane-1,4,7,10-tetraacetic acid (DOTA) derivatives for chemoselective attachment to unprotected polyfunctionalized compounds. *Chemistry* **2007**, *13*, 6082-6090, doi:10.1002/chem.200700231.
6. Theodossiou, T.A.; Pantos, A.; Tsogas, I.; Paleos, C.M. Guanidinylated dendritic molecular transporters: prospective drug delivery systems and application in cell transfection. *ChemMedChem* **2008**, *3*, 1635-1643, doi:10.1002/cmdc.200800190.
7. Feichtinger, K.; Zapf, C.; Sings, H.L.; Goodman, M. Diprotected Triflylguanidines: A New Class of Guanidinylation Reagents. *The Journal of Organic Chemistry* **1998**, *63*, 3804-3805, doi:10.1021/jo980425s.
