# Supplementary material for: Synergistic Suppression of NF1 Malignant Peripheral Nerve Sheath Tumor Cell Growth in Culture and Orthotopic Xenografts by Combinational Treatment with Statin and Prodrug Farnesyltransferase Inhibitor PAMAM G4 Dendrimers
Source: Cancers (Basel). 2023 Dec 23;16(1):89. doi: 10.3390/cancers16010089 (PMC10778372; doi:10.3390/cancers16010089)

Figure S11. Photographs and MRI analyses of S462TY sciatic nerve xenograph growing in NOD SKID mouse

Saline Control Treatment, image 47 day post tumor injection
Tumor Volume = 25.6 mm^3

Reconstituted MRI Slices

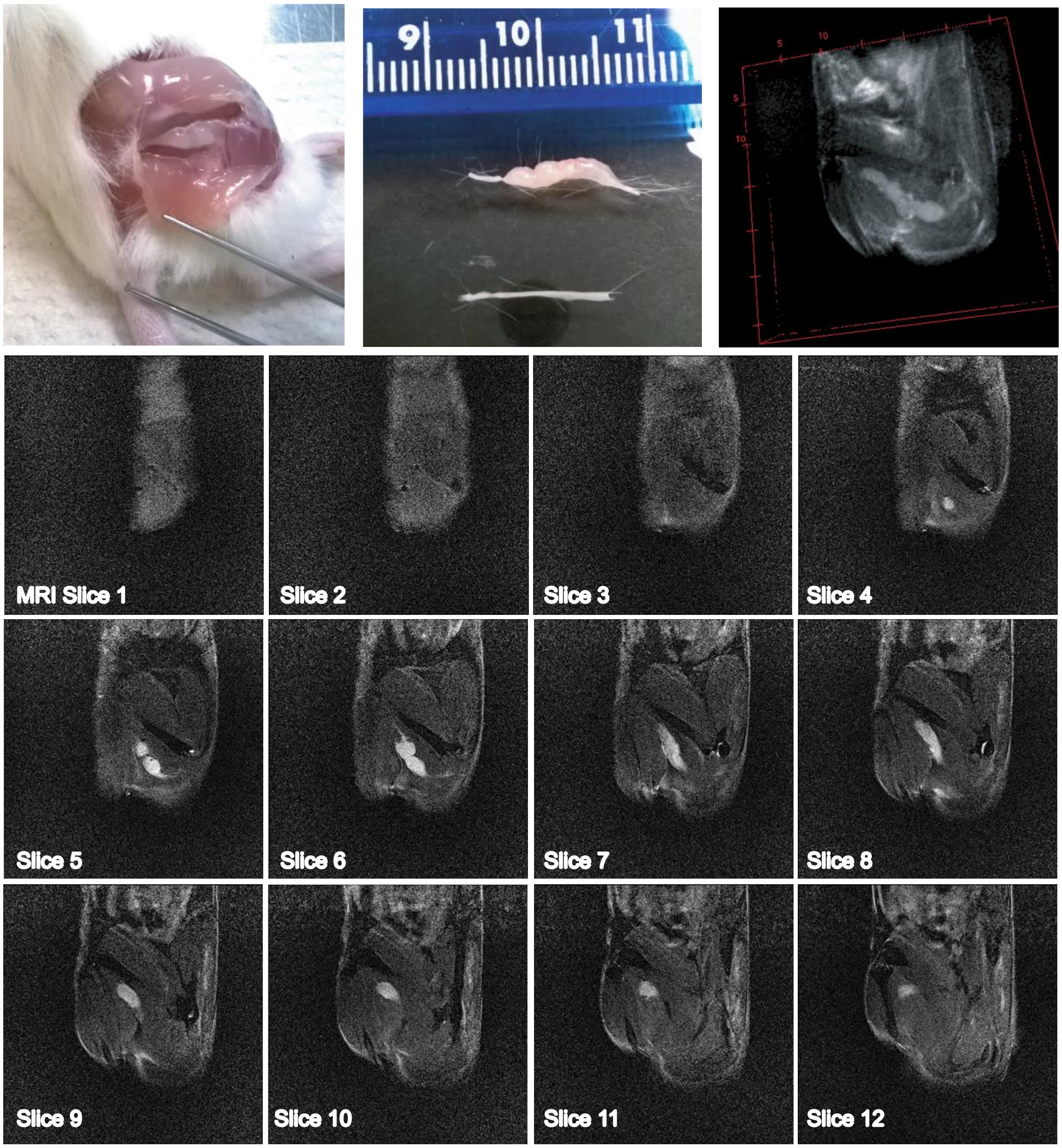

Supplement: Supplementary file 1 [file cancers-16-00089-s001.zip › Figure S11.pdf]
