# Supplementary material for: Synergistic Suppression of NF1 Malignant Peripheral Nerve Sheath Tumor Cell Growth in Culture and Orthotopic Xenografts by Combinational Treatment with Statin and Prodrug Farnesyltransferase Inhibitor PAMAM G4 Dendrimers
Source: Cancers (Basel). 2023 Dec 23;16(1):89. doi: 10.3390/cancers16010089 (PMC10778372; doi:10.3390/cancers16010089)
Supplement: Supplementary file 1 [file cancers-16-00089-s001.zip › Figure S12.pdf]

Figure S12. Synergistic inhibition of non-small cell carcinoma A549 colony formation by combinational lovastatin and IG 2 treatment

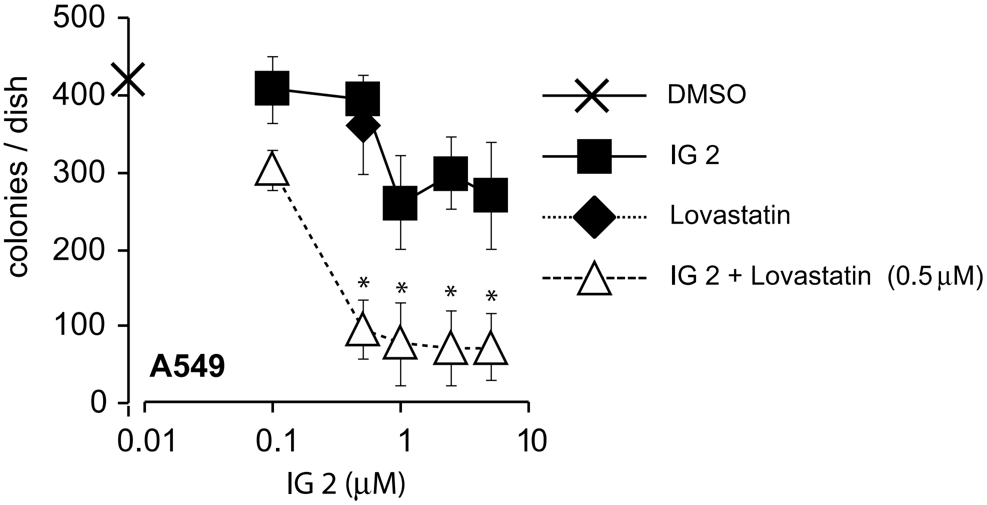

Figure S12. Synergistic inhibition of non-small cell carcinoma A549 colony formation by combinational lovastatin and **IG 2** treatment. Overnight cultures of A549 cells were treated with DMSO, 0.5 μM lovastatin, or varied concentrations of prodrug **IG 2**. After four days of treatment the cultures were washed and refed with fresh medium lacking any drugs. Cultures were fixed and stained 5 days later for assessment of colony formation. Treatments are noted in the figure. All data represent means ± SD of 4 plates per treatment group. *Significantly less than colonies in cultures treated with DMSO, 0.5 μM lovastatin or **IG 2** alone.
