# Supplementary material for: Synergistic Suppression of NF1 Malignant Peripheral Nerve Sheath Tumor Cell Growth in Culture and Orthotopic Xenografts by Combinational Treatment with Statin and Prodrug Farnesyltransferase Inhibitor PAMAM G4 Dendrimers
Source: Cancers (Basel). 2023 Dec 23;16(1):89. doi: 10.3390/cancers16010089 (PMC10778372; doi:10.3390/cancers16010089)

## Figure S3. Whole gel images of Rab5A and GAPDH blots used to compose Figure 7B

### Antibodies are to Rab5A (top blot) and to GAPDH loading control (bottom blot)

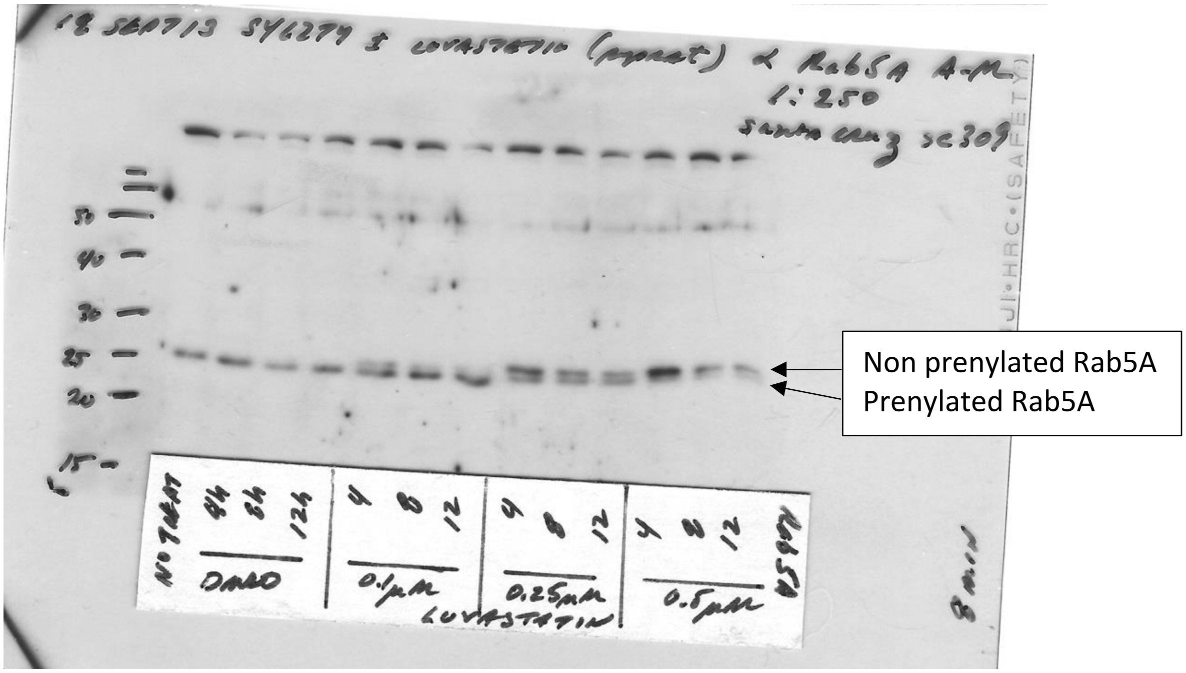


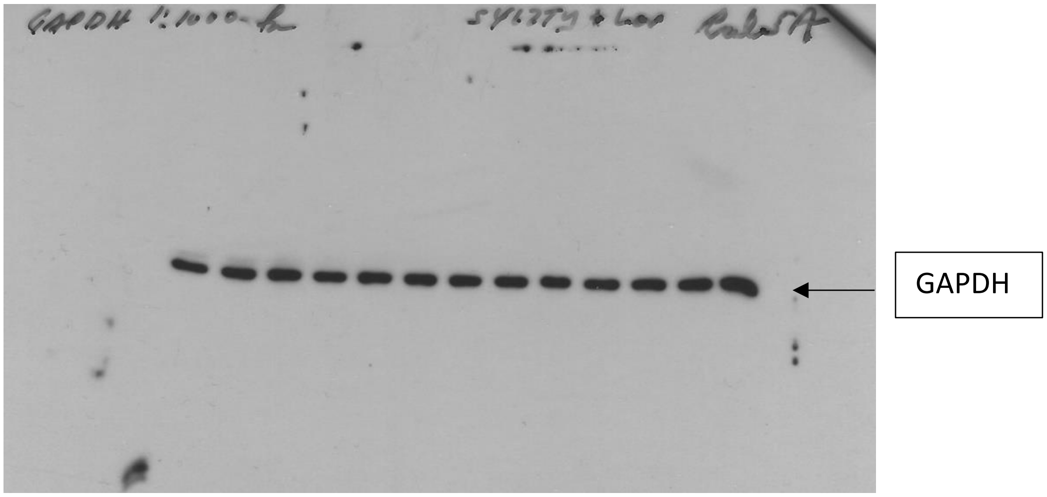

Supplement: Supplementary file 1 [file cancers-16-00089-s001.zip › Figure S3.pdf]
