# Supplementary material for: Synergistic Suppression of NF1 Malignant Peripheral Nerve Sheath Tumor Cell Growth in Culture and Orthotopic Xenografts by Combinational Treatment with Statin and Prodrug Farnesyltransferase Inhibitor PAMAM G4 Dendrimers
Source: Cancers (Basel). 2023 Dec 23;16(1):89. doi: 10.3390/cancers16010089 (PMC10778372; doi:10.3390/cancers16010089)

Figure S8. Whole gel images of pan Ras and GAPDH blots used to compose Figure 8B

Note: ib21-81 is another name for IG 2

Antibodies are to pan Ras (top blot) and to GAPDH loading control (bottom blot)

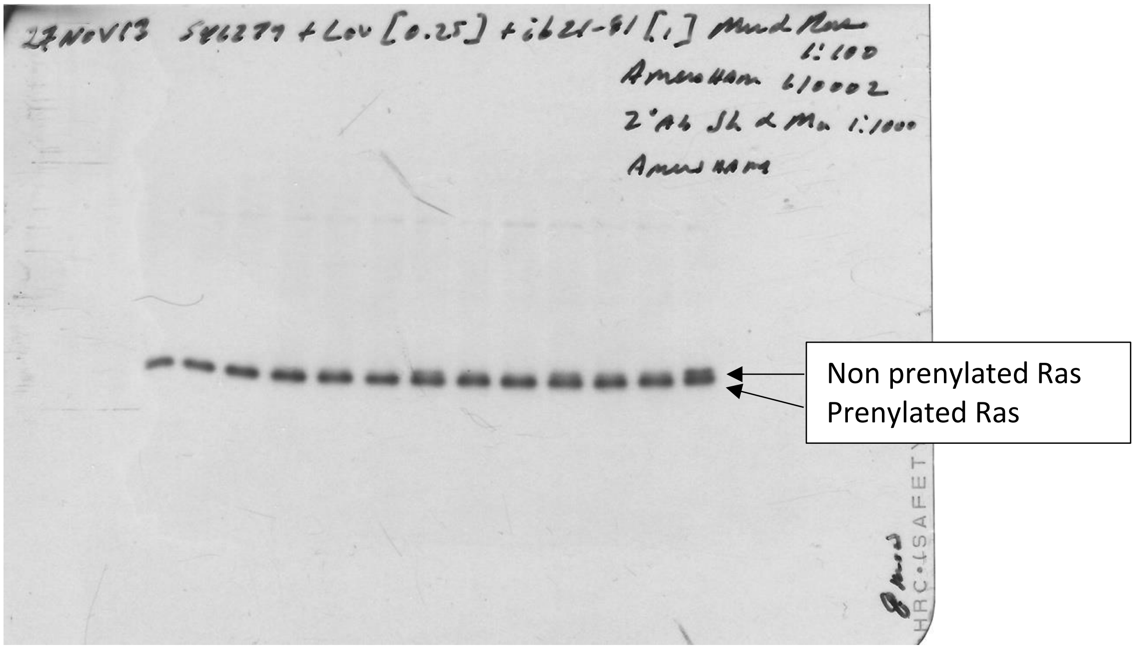

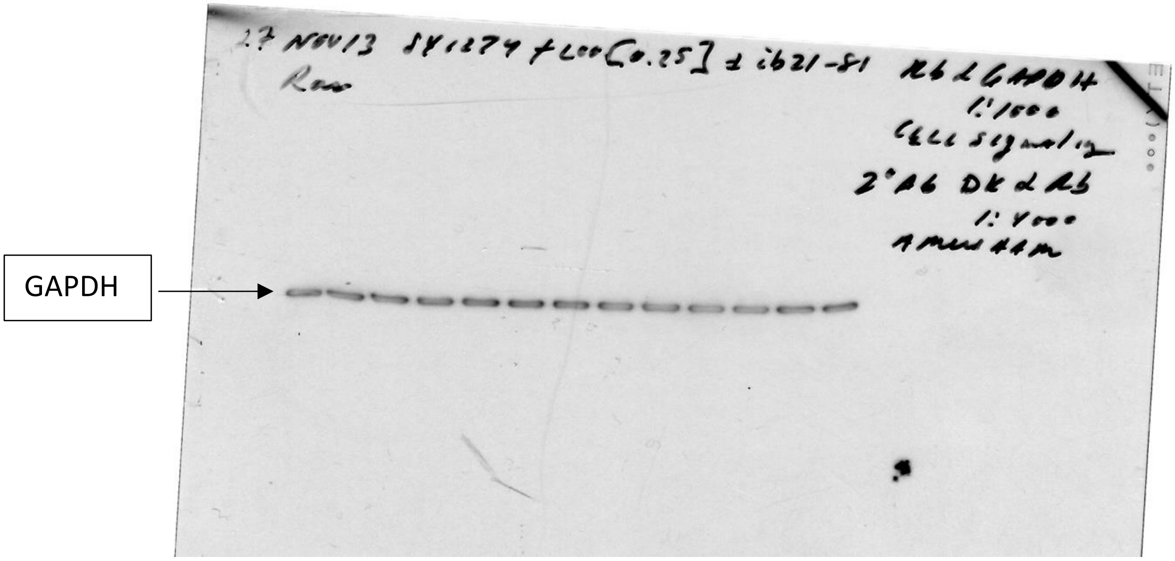

Supplement: Supplementary file 1 [file cancers-16-00089-s001.zip › Figure S8.pdf]
