# Supplementary material for: Synergistic Suppression of NF1 Malignant Peripheral Nerve Sheath Tumor Cell Growth in Culture and Orthotopic Xenografts by Combinational Treatment with Statin and Prodrug Farnesyltransferase Inhibitor PAMAM G4 Dendrimers
Source: Cancers (Basel). 2023 Dec 23;16(1):89. doi: 10.3390/cancers16010089 (PMC10778372; doi:10.3390/cancers16010089)
Supplement: Supplementary file 1 [file cancers-16-00089-s001.zip › List of Supplementary materials.pdf]

List of Supplementary Materials:

Methods for FTI and FTI Dendrimer Syntheses, includes Scheme S1
Figure S1. Whole gel images of Rab5A and GAPDH blots used to compose Figure 7A
Figure S2. Whole gel images of Rap1A and actin blots used to compose Figure 7A
Figure S3. Whole gel images of Rab5A and GAPDH blots used to compose Figure 7B
Figure S4. Whole gel images of Rap1A and actin blots used to compose Figure 7B
Figure S5. Whole gel images of Rab5A and GAPDH blots used to compose Figure 8A
Figure S6. Whole gel images of Rap1A and GAPDH blots used to compose Figure 8A
Figure S7. Whole gel images of Rap1A and GAPDH blots used to compose Figure 8B
Figure S8. Whole gel images of pan Ras and GAPDH blots used to compose Figure 8B
Figure S9. Whole gel images of N-Ras and GAPDH blots used to compose Figure 8C
Figure S10. Whole gel images of N-Ras and GAPDH blots used to compose Figure 9
Figure S11. Photographs and MRI analyses of S462TY sciatic nerve xenograph growing in NOD SKID mouse
Figure S12. Synergistic inhibition of non-small cell carcinoma A549 colony formation by combinational lovastatin and IG 2 treatment
